# Robust Analysis of Fluxes in Genome-Scale Metabolic Pathways

**DOI:** 10.1038/s41598-017-00170-3

**Published:** 2017-03-21

**Authors:** Michael MacGillivray, Amy Ko, Emily Gruber, Miranda Sawyer, Eivind Almaas, Allen Holder

**Affiliations:** 1 Department of Mathematics, Rose-Hulman Institute of Technology, Terre Haute, IN USA; 2 Department of Biotechnology, NTNU - Norwegian University of Science and Technology, Trondheim, Norway; 3 K.G. Jebsen Center for Genetic Epidemiology, Department of Public Health and General Practice, NTNU - Norwegian University of Science and Technology, Trondheim, Norway

## Abstract

Constraint-based optimization, such as flux balance analysis (FBA), has become a standard systems-biology computational method to study cellular metabolisms that are assumed to be in a steady state of optimal growth. The methods are based on optimization while assuming (i) equilibrium of a linear system of ordinary differential equations, and (ii) deterministic data. However, the steady-state assumption is biologically imperfect, and several key stoichiometric coefficients are experimentally inferred from situations of inherent variation. We propose an approach that explicitly acknowledges heterogeneity and conducts a robust analysis of metabolic pathways (RAMP). The basic assumption of steady state is relaxed, and we model the innate heterogeneity of cells probabilistically. Our mathematical study of the stochastic problem shows that FBA is a limiting case of our RAMP method. Moreover, RAMP has the properties that: A) metabolic states are (Lipschitz) continuous with regards to the probabilistic modeling parameters, B) convergent metabolic states are solutions to the deterministic FBA paradigm as the stochastic elements dissipate, and C) RAMP can identify biologically tolerable diversity of a metabolic network in an optimized culture. We benchmark RAMP against traditional FBA on genome-scale metabolic reconstructed models of *E*. *coli*, calculating essential genes and comparing with experimental flux data.

## Introduction

Constraint-based analysis is an approach to study metabolic networks that has become a primary computational tool with a well established literature^1–3^. The foundation of constraint-based analysis is a representation of a cell’s metabolism as a linear system of differential equations in the (unknown) fluxes of the metabolic reactions. The additional assumption that the metabolic network has reached steady state results in a homogeneous system. It is customary to optimize an appropriate objective over the fluxes that achieve steady state to model how a cellular metabolism reacts as it is held in a constant environment^4, 5^. Different objectives have been proposed and studied^6, 7^, with the most prevalent being the maximization of the rate at which biomass is created.

The most commonly applied version of constraint-based analysis is known as flux balance analysis (FBA). The original study of FBA investigated small portions of the central metabolism^8^, but the use of constraint-based methods has expanded to encompass genome-scale metabolic models and a diverse range of applications and computational formulations^9^. However, the basic premises of optimality and steady state have remained intact, although these assumptions are well known to be inexact from a biochemistry perspective. That said, there are two somewhat evident reasons to leave these premises unchecked. First, the resulting optimization problems are most often linear and less often quadratic (convex), and in both cases the problems are easily solved with standard methods and software. Second, traditional constraint-based models are useful even with questionable premises. For example, with a high level of accuracy, FBA models can identify essential genes^10^, predict metabolic responses as pathways are interrupted^11^, determine central metabolic pathways^12–14^, develop synthetic biology engineering strategies^15–18^, as well as identify genes showing promise as inhibitors of cancer migration^19^. Hence, a justified retort by the constraint-based analysis community to those who might question the two defining premises is that the modeling approach yields meaningful and computationally tractable science even though the cornerstone premises are imperfect.

The traditional constraint-based paradigm assumes a model of an individual cell that has reached an ideal, limiting setting of steady state. Models are benchmarked with experimental outcomes under the assumption that experimental measurements are conducted on a population of identical cells that have evolved to optimal performance. However, experimental reality differs from this assumption. First, even well optimized cellular populations of identical cells exhibit prominent heterogeneity in uptake, secretion, and growth rates^4^. Second, experimental flux measurements constitute averages over such heterogeneous populations. Moreover, these averages provide the only possible experimental prototype of cellular flux since single cell measurements are currently impossible. Third, heterogeneity of metabolic rates is innate within a culture because cells vary within, e.g., their cell cycles and replication states. The traditional paradigm that a constraint-based model represents a single, optimized cell is certainly challenged by such experimental realities upon which models are vetted.

Here, we challenge and relax the foundational assumption of a system being in a steady state, and we instead introduce a robust optimization^20^ counterpart to FBA, called Robust Analysis of Metabolic Pathways (RAMP). The robust optimization implementation allows us to model a system stochastically where, instead of imposing the rigid and simplistic condition of deterministic coefficients and steady state, we allow these assumptions to be relaxed. In RAMP we control departures from steady state by limiting their *likelihood of deviation*. Since the stochastic flexibility of RAMP permits us to postulate metabolic models for cultures that deviate from steady state, this opens the possibility of computationally studying functional states of cellular metabolisms as they transition toward a steady state. Such an investigation begs the questions of (1) whether or not RAMP solutions are continuous in their stochastic elements and (2) whether or not convergent trajectories of RAMP solutions yield deterministic FBA solutions as the probabilistic assumptions abate. We mathematically answer both questions in the affirmative, and hence, FBA is justly thought of as the limit of evolutionary adaptations of a cellular metabolic network that achieves steady state.

Additionally, the development and stochastic formulation of RAMP explicitly allows us to systematically address heterogeneity in metabolic phenotypes that exists in isogenic cellular populations^21–23^. Our mathematical analysis identifies the probabilistic situations that coincide with our modeling framework once the limiting deterministic setting is reached. Consequently, if we assume that RAMP’s modeling paradigm aligns with the stochastic nature of a culture, then we can mathematically characterize the ways in which a culture may harbor variation.

Possible concerns with RAMP are that it could lose the predictive successes of FBA, and that it could be less computationally tractable. We have designed our computational experiments to compare RAMP with FBA in such a way that both models can be solved with the default simplex algorithms of several linear solvers, and our results on two *E*. *coli* models^24, 25^ demonstrate that RAMP rivals FBA in its ability to identify essential genes. We further show that RAMP’s efficacy in identifying essential genes is predominantly stable as individual coefficients of the biomass equation are assumed to be uncertain. The ranges over which individual coefficients can vary are disparate, having a surprising spread of many orders of magnitude. All coefficients could accommodate uncertainty of at least 0.42%, with most extending beyond 100% uncertainty. We also compare FBA’s and RAMP’s consistency with experimentally determined fluxes^6, 26, 27^, finding that RAMP significantly outperforms FBA for both aerobic and anaerobic conditions.

With regards to computational tractability, RAMP is a robust linear program, which is a second-order cone program (SOCP) that is known to be solvable in polynomial time^28, 29^. Such robust models were developed to overcome problems with over-optimizing designs, meaning that a design could be optimal with respect to the estimated data on which it was built but (much) less so as the data varied over realistic possibilities^30^. A similar concern about over-optimization has been expressed in the FBA literature^7^, and RAMP directly addresses this issue by expressing variability within the model itself.

We note that an alternative robust model has been suggested^31^. This method differs significantly from RAMP since it uses a robust least squares method in a bi-objective model. Such a model can illustrate the trade-off between the optimal growth rate and the deviation from steady state, whereas RAMP uses robust linear programming. Furthermore, the numerical work in ref. 31 is conducted on a small, illustrative example, and there is no extrapolation of the bi-objective model to a realistic genome-scale metabolic reconstruction.

Our conclusion is that RAMP opens a new paradigm for constraint-based optimization and analysis of genome-scale metabolic models that is also directly applicable to a large number of the recent constraint-based extensions to FBA. Our computational implementation and benchmarking of a specific version of RAMP using both linear and non-nonlinear objective functions, show that it is a computationally tractable alternative and complement to traditional FBA.

## Results

We begin by re-visiting the foundational assumptions of FBA, where the metabolism of an individual cell *c* is defined by a system of *n* reactions, with the *j*-th reaction being

$${a}_{1j}^{c}[{x}_{1}^{c}]+{a}_{2j}^{c}[{x}_{2}^{c}]+\ldots +{a}_{mj}^{c}[{x}_{m}^{c}]\underset{{k}_{-}^{cj}}{\overset{{k}_{+}^{cj}}{\rightleftharpoons }}{b}_{1j}^{c}[{x}_{1}^{c}]+{b}_{2j}^{c}[{x}_{2}^{c}]+\ldots +{b}_{mj}^{c}[{x}_{m}^{c}].$$

Here, $[{x}_{i}^{c}]$ denotes the concentration of the *i*-th metabolite in cell *c*. The cellular superscript *c* is absent in FBA’s standard development due to the assumption that an average cell is being studied. Thus, the ordinary differential equation for the *i*-th metabolic concentration in cell *c* is,

1 $$\begin{array}{rcl}\frac{d[{x}_{i}^{c}]}{dt} & = & \sum _{j=1}^{n}({b}_{ij}^{c}-{a}_{ij}^{c})({k}_{+}^{cj}\,{[{x}_{1}^{c}]}^{{a}_{1j}^{c}}{[{x}_{2}^{c}]}^{{a}_{2j}^{c}}\ldots {[{x}_{m}^{c}]}^{{a}_{mj}^{c}}-{k}_{-}^{cj}{[{x}_{1}^{c}]}^{{b}_{1j}}{[{x}_{2}^{c}]}^{{b}_{2j}}\ldots {[{x}_{m}^{c}]}^{{b}_{mj}})\\  & = & \sum _{j=1}^{n}{S}_{ij}^{c}{v}_{j}^{c},\end{array}$$

where ${S}_{ij}^{c}$ is the stoichiometric coefficient for metabolite *i* in reaction *j*, the flux of reaction *j* is ${v}_{j}^{c}$, and *c* denotes the cell. Consequently, genetically identical individuals of a culture share the same collection of metabolic reactions. Hence, the preponderance of stoichiometric coefficients of any particular genome-scale metabolic reconstruction are static across the individuals of a culture. However, a complete metabolic model includes several inferred and less certain reactions to model processes, such as those involved in the creation of biomass. In many of the recent extensions of FBA^9^, a combination of alternative objective functions, global constraints or upper/lower bounds on sets of reaction fluxes are added to the system. Consequently, many extensions, such as FBAwMC^32^, GIMME^33^, MOMENT^34^, and GX-FBA^35^, are implemented by adding rows and/or columns to the FBA stoichiometric matrix, and these new stoichiometric coefficients do not generally consist of integer values. Instead, depending on the method of interest, these added stoichiometric coefficients may consist of a combination of data from large-scale’ omics sources, biochemistry knowledge, or theoretical modelling assumptions. Thus, they are carriers of inherent uncertainty.

In particular, the experimentally determined coefficients of a biomass equation are noticeably different from their integral counterparts in the shared metabolism, see Table 1 as an example from the *E*. *coli* metabolic model iJR904^36^. These less certain coefficients would be stochastic, time varying parameters per individual cell. Thus, instead of the growth reaction in Table 1 being imposed on all cells in a population, these coefficients should be able to vary among the individual cells.

**Table 1:** An example of the non-integer coefficients, in parentheses, of the input and output metabolites of the *E*. *coli* metabolic model iJR904 growth reaction^36^.

Input Metabolites			
ACCOA(−0.00005)	ALA(−0.488)	AMP(−0.001)	ARG(−0.281)
ASN(−0.229)	ASP(−0.229)	ATP(−45.73)	CL(−0.00645)
COA(−0.000006)	CTP(−0.126)	CYS(−0.087)	DATP(−0.0247)
DCTP(−0.0254)	DGTP(−0.0254)	DTTP(−0.0247)	FAD(−0.00001)
GLN(−0.25)	GLU(−0.25)	GLY(−0.582)	GLYCOGEN(−0.154)
GTP(−0.203)	HIS(−0.09)	ILE(−0.276)	LEU(−0.428)
LPS(−0.0084)	LYS(−0.326)	MET(−0.146)	MTHF(−0.05)
NAD(−0.00215)	NADH(−0.00005)	NADP(−0.00013)	NADPH(−0.0004)
PE(−0.09675)	PEPTIDO(−0.0276)	PG(−0.02322)	PHE(−0.176)
PRO(−0.21)	PS(−0.00258)	PTRC(−0.035)	SER(−0.205)
SPMD(−0.007)	SUCCOA(−0.000003)	THR(−0.241)	TRP(−0.054)
TYR(−0.131)	UDPG(−0.003)	UTP(−0.136)	VAL(−0.402)
↓			
ADP(45.560000)	Biomass(1.000000)	PI(45.560000)	PPI(0.730200)
Output Metabolites			

There exists also other sources of uncertainty for the performance of a genome-scale metabolic reconstruction, such as the value of the phosphate/oxygen (P/O) ratio. Changes in how the network performs the ATP production and drain has a significant potential to affect model-predicted growth performance. In the following discussion, however, we will base our presentation and discussion on uncertainty in the biomass growth coefficients.

Suppose $\hat{c}$ indexes the single, average cell assumed by FBA. The limiting steady state assumption of FBA constrains the fluxes so that,

$$\frac{d[{x}_{i}^{\hat{c}}]}{dt}=\sum _{j=1}^{n}{S}_{ij}^{\hat{c}}{v}_{j}^{\hat{c}}=\mathrm{0,}\,\,\forall i,$$

which is the equilibrium condition as *t* → ∞. Furthermore, it has been suggested that cellular metabolic performance evolves toward an optimal growth state as a culture’s environment is left unchanged and as populations are repeatedly sampled and re-grown^4, 10^. FBA is predicated on the assumption that cells have been tuned through such an evolutionary processes to an “optimal” use of their resources, where “optimal” is measured by a function of the fluxes, *g*(*v*). Hence, FBA studies metabolic processes through optimization problems, and adaptations thereof, of the form

2 $${\rm{\max }}\{g({v}^{\hat{c}})\,:{S}^{\hat{c}}{v}^{\hat{c}}=0,{L}^{\hat{c}}\le {v}^{\hat{c}}\le {U}^{\hat{c}}\},$$

where ${S}^{\hat{c}}$ is the stoichiometric matrix whose components are ${S}_{ij}^{\hat{c}}$ and ${v}^{\hat{c}}$ is the associated (decision) vector of fluxes. The objective function *g*(*v*) is typically chosen to estimate the rate at which biomass is created, and for convenience, we assume throughout that *g*(*v*) is the sole flux of the growth reaction, i.e. *g*(*v*) = *v*_Growth_. While several alternative objectives have been proposed and investigated in different biological settings^6, 7^, the production of biomass is the most prevalent choice and is the default in the COBRA^37^ toolbox. The vectors of lower bounds, ${L}^{\hat{c}}$, and upper bounds, ${U}^{\hat{c}}$, may contain ±∞, or some suitably large value, to indicate that a flux is unbounded. If ${L}_{j}^{\hat{c}} < 0$ and ${U}_{j}^{\hat{c}} > 0$, then reaction *j* is reversible.

### A probabilistic development of RAMP

We assume that a culture has gone through a long-enough time of growth and re-sampling to be largely optimized to the resources of its (invariant) environment. Heterogeneity within the culture is modeled as the random experiment that is the growth of the culture, and we consider the variational states of the genetically identical cells of the culture as independent and identically distributed outcomes from that growth experiment. Hence, the known heterogeneity within an optimized culture is the result of a large sample of possible outcomes. Consequently, for each metabolite *i* the collection of $d[{x}_{i}^{c}]/dt$ across the cells *c* of the culture is a random sample of the stochastic rate of change of the concentration. Let ${\mathscr{C}}$ be the collection of cells in the culture and $d[{x}_{i}^{{\mathscr{C}}}]/dt$ be the average rate of change of the concentration (of metabolite *i*) over the sample, that is

$$\frac{d[{x}_{i}^{{\mathscr{C}}}]}{dt}=\frac{1}{|{\mathscr{C}}|}\sum _{c\in {\bf{C}}}\frac{d[{x}_{i}^{c}]}{dt}.$$

The central limit theorem mandates that $d[{x}_{i}^{{\mathscr{C}}}]/dt$ be approximately normal, and we let *μ*_*i*_ and *σ*_*i*_ be the unknown mean and standard deviation of this normal distribution. This approximation is expected to be trustworthy since cultures regularly contain hundreds of millions of cells.

We replace the traditional static assumption of steady state with the probability constraints

3 $$P(d[{x}_{i}^{{\mathscr{C}}}]/dt > {M}_{i})\le \varepsilon \quad {\rm{and}}\quad P(d[{x}_{i}^{{\mathscr{C}}}]/dt < -{M}_{i})\le \varepsilon ,$$

which combine to ensure that $P(-{M}_{i}\le d[{x}_{i}^{{\mathscr{C}}}]/dt\le {M}_{i})\ge 1-2\varepsilon$. Here, |*M*_*i*_| is a bound on the feasible fluxes that define the allowed deviation from steady state. Unlike the FBA model formulation that collapses onto an ideal cell and ignores cultural variations, these stochastic constraints account for the innate variations that are known to exist. Since $(d[{x}_{i}^{{\mathscr{C}}}]/dt-{\mu }_{i})/{\sigma }_{i}$ is a standard normal variable, we write the constraints in Eq. (3) as

$$\frac{{M}_{i}-{\mu }_{i}}{{\sigma }_{i}}\ge {\delta }_{1-\varepsilon }\quad {\rm{and}}\,\,\frac{-{M}_{i}-{\mu }_{i}}{{\sigma }_{i}}\le {\delta }_{\varepsilon },$$

where *δ*_*ε*_ and *δ*_1−*ε*_ are the *ε* and 1 − *ε* percentiles respectively, i.e. $P((d[{x}_{i}^{{\mathscr{C}}}]/dt-{\mu }_{i})/{\sigma }_{i} > {\delta }_{1-\varepsilon })=\varepsilon$. Rearranging these inequalities and using the fact that *δ*_1−*ε*_ = −*δ*_*ε*_, we find

4 $${\sigma }_{i}\le \,{\rm{\min }}\{\frac{{M}_{i}-{\mu }_{i}}{{\delta }_{1-\varepsilon }},\frac{{M}_{i}+{\mu }_{i}}{{\delta }_{1-\varepsilon }}\}.$$

The stochastic constraints in Eq. (4) are expressed in terms of the fluxes by extending the static differential equation of an individual cell in Eq. (1) to the stochastic differential equation of a culture. The standard assumption has been that the ideal cell of an FBA model is an average representation that matches measurements of a culture. If we allow *S*_*i*_ to be the *i*-th row of the random stoichiometric matrix in which the uncertain elements are random variables, e.g. for the creation of biomass, then the implicit FBA assumption of an ideal cell results in the following stochastic differential equation for each metabolite,

5 $$\frac{d[{x}_{i}^{\hat{c}}]}{dt}=\frac{d[{x}_{i}^{{\mathscr{C}}}]}{dt}={S}_{i}v.$$

Consequently, for any flux vector *v* we have

$${\mu }_{i}=E(d[{x}_{i}^{\hat{c}}]/dt)=E(d[{x}_{i}^{{\mathscr{C}}}]/dt)=E({S}_{i}v)=E({S}_{i})v.$$

An apt interpretation of FBA’s projection of a culture’s heterogeneity onto an average is somewhat laid bare by these equalities: FBA assumes that *μ*_*i*_ = 0. The first and second equalities then constrain the expectation of the sample mean, $d[{x}_{i}^{{\mathscr{C}}}]/dt$ to be zero. The ideal FBA cell $\hat{c}$ is interpreted as a stochastic representation of the culture; a representation that inherits the expected averages of the metabolic rates of change over a culture. The FBA paradigm thus acknowledges, but ignores within its modeling framework, deviation from steady state since FBA only insists that the expectation be zero without regard to variation. The third equality is a direct consequence of Eq. (5), and the last equality follows from the linearity of the expectation. Hence FBA assumes *E*(*S*_*i*_)*v* = 0, which shows that the stoichiometric coefficients of an FBA model are reasonably interpreted as averages, i.e. that ${S}_{i}^{\hat{c}}=E({S}_{i})$. Hence, growth coefficients, such as those in Table 1, are expectations attributed to an ideal cell in hopes of representing an entire culture. If a stoichiometric coefficient is certain because it is part of the metabolic network that is common to the taxa, then the average is simply the known value that is shared among all cells. If the coefficient is otherwise uncertain and assumed to be random, then FBA assumes the mean value.

The equation *E*(*S*_*i*_)*v* = *μ*_*i*_ shows how the vector of fluxes *v* defines the expected value of the rate of change of the concentration (of metabolite *i*), and the equation begs for detailed random models of the uncertain stoichiometric coefficients. Such detail is challenged by experimental limitations. For example, it is impossible to study individual cells or even groups of cells that are in identical metabolic or proteomic states. Hence, inferring accurate distributional characteristics on parameters such as the growth coefficients is currently impossible. Beyond averages, our ability to express Eq. (4) in terms of the fluxes further necessitates the calculation of standard deviations.

#### Scenario analysis in RAMP

A common statistical supposition is to impose additional requirements. For example, we could outright assume that the individual random elements of *S*_*i*_ are independent and identically distributed. However, such a requirement would be dubious in our setting due to the certain dependence among growth and metabolic uptake and excretion. We instead promote the use of scenario analysis to query the behavior of the stochastic model. Scenarios have been used in other application domains in which similar optimization problems arise. For instance, scenarios are motivated even with an assumption of independent and identically distributed random variables to better manage computational space^38^. In addition to overcoming our inability to accurately model the separate random stoichiometric coefficients, scenarios have the practical advantage of being easily interpreted as biological possibilities. Importantly, the normality of $d[{x}_{i}^{{\mathscr{C}}}]/dt$ is independent of the distributions used to model the uncertain stoichiometric coefficients. The scenarios in RAMP’s development and mathematical analysis are purposely arbitrary so that RAMP remains viable no matter which (discrete) distributions might actually model a biochemical reality.

Assuming that each *S*_*i*_ has *q* random scenarios, we let *S*_*ik*_ be the (non-random) stoichiometric coefficients for the *i*-th metabolite in scenario *k* and let *p*_*ik*_ be the probability of scenario *k*, for *k* = 1, 2, 3, …, *q*. Allowing *p*_*i*_ to be the (positive) vector of probabilities indexed by *k*, *P*_*i*_ to be the (positive definite) diagonal matrix formed by *p*_*i*_, and ${\hat{S}}_{i}$ to be the matrix whose *k*-th row is *S*_*ik*_, the mean and variance of *S*_*i*_ *v* are

6 $${\mu }_{i}={\rm{E}}({S}_{i}v)={\rm{E}}({S}_{i})v=\sum _{k=1}^{q}{p}_{ik}{S}_{ik}v={p}_{i}^{T}{\hat{S}}_{i}v$$

and

$${\sigma }_{i}^{2}={\rm{Var}}({S}_{i}v)=\sum _{k=1}^{q}{p}_{ik}{({S}_{ik}v-{\rm{E}}({S}_{ik}v))}^{2}={({\hat{S}}_{i}v)}^{T}{(I-e{p}_{i}^{T})}^{T}{P}_{i}(I-e{p}_{i}^{T}){\hat{S}}_{i}v,$$

where *e* is a vector of ones. Defining ${R}_{i}={\delta }_{1-\varepsilon }\sqrt{{P}_{i}}(I-e{p}_{i}^{T}){\hat{S}}_{i}$, we have that the standard deviation is

7 $${\sigma }_{i}=\sqrt{{\rm{Var}}({S}_{i}v)}=\sqrt{{\Vert {R}_{i}v\Vert }^{2}/{\delta }_{1-\varepsilon }^{2}}=\Vert {R}_{i}v\Vert /{\delta }_{1-\varepsilon }.$$

The expected value and standard deviation are related through constraint (4), from which we have

$$\Vert {R}_{i}v\Vert \le \,{\rm{\min }}\{{M}_{i}-{p}_{i}^{T}{\hat{S}}_{i}v,{M}_{i}+{p}_{i}^{T}{\hat{S}}_{i}v\},$$

or equivalently,

8 $$\Vert {R}_{i}v\Vert -{M}_{i}\le {p}_{i}^{T}{\hat{S}}_{i}v\le {M}_{i}-\Vert {R}_{i}v\Vert .$$

Although Eq. (8) is more complicated than a traditional linear constraint, it is the combination of two second order cone constraints. Such constraints share several desirable properties with linear constraints, such as being convex. Replacing the traditional linear constraints ${S}^{\hat{c}}v=0$ with Eq. (8), we have arrived at an SOCP that is our RAMP model:

9 $$\begin{array}{c}{\rm{\max }}\,{v}_{{\rm{Growth}}}\\ {\rm{subject}}\,{\rm{to}}\\ \Vert {R}_{i}v\Vert -{M}_{i}\le {p}_{i}^{T}{\hat{S}}_{i}v\le {M}_{i}-\Vert {R}_{i}v\Vert ,\quad i=\mathrm{1,\; 2},\ldots ,m\\ L\le v\le U.\end{array}\}$$

The RAMP model defaults to a commonality of the upper and lower flux bounds among the cells so that *L* ^*c*^ = *L* and *U* ^*c*^ = *U* for all *c*. This default is somewhat for notational convenience since stochastic variation in *L* or *U* could be remodeled as variation in the coefficient of the associated flux, and the resulting constraint would then be part of the stoichiometric matrix^20^.

A feasible RAMP solution is a collection of fluxes under which the mean and variance of the normal distribution of $d[{x}_{i}^{{\mathscr{C}}}]/dt$ satisfies the likelihood of being in steady state with regard to the spectrum of stoichiometric possibilities. A mathematical expression of this fact is possible upon recognizing that

$$\Vert {R}_{i}v\Vert =\,{\rm{\max }}\,\{{u}^{T}{R}_{i}v\,:\Vert u\Vert \le 1\},$$

which allows the right-hand side of the SOCP constraint to be re-written as

10 $$\begin{array}{rcl}{p}_{i}^{T}{\hat{S}}_{i}v+\Vert {R}_{i}v\Vert \le {M}_{i} & \iff  & {p}_{i}^{T}{\hat{S}}_{i}v+\,{\rm{\max }}\{{u}^{T}{R}_{i}v\,:\Vert u\Vert \le 1\}\le {M}_{i}\\  & \iff  & {\rm{\max }}\{{p}_{i}^{T}{\hat{S}}_{i}v+{u}^{T}{R}_{i}v\,:\Vert u\Vert \le 1\}\le {M}_{i}\\  & \iff  & {S}_{i}v\le {M}_{i},\forall {S}_{i}\in \{{p}_{i}^{T}{\hat{S}}_{i}+{u}^{T}{R}_{i}\,:\Vert u\Vert \le 1\}.\end{array}$$

The left-hand side SOCP constraint can be similarly re-written as,

11 $$-{p}_{i}^{T}{\hat{S}}_{i}v+\Vert {R}_{i}v\Vert \le {M}_{i}\iff {S}_{i}v\le {M}_{i}\forall {S}_{i}\in \{-{p}_{i}^{T}{\hat{S}}_{i}+{u}^{T}{R}_{i}\,:\Vert u\Vert \le 1\}.$$

The sets from which *S*_*i*_ are drawn are called uncertainty sets in the robust optimization literature, and these sets define the collection of stoichiometric possibility. A feasible flux must satisfy being within |*M*_*i*_| of steady state for all possible stoichiometric possibilities, meaning that *S*_*i*_ can deviate from its average ${p}_{i}^{T}{\hat{S}}_{i}$ by as much as *u* ^*T*^ *R*_*i*_ as long as $\Vert u\Vert \le 1$.

#### Illustrative example of the RAMP method

The re-expression of the SOCP constraints in terms of their uncertainty sets provides a geometric description. As an illustrative example, consider the two variable system

$${S}_{1}v={s}_{1}{v}_{1}+{s}_{2}{v}_{2}=\mathrm{0,}-1\le {v}_{1}\le \mathrm{1,}-1\le {v}_{2}\le \mathrm{1,}$$

where the average values are *s*_1_ = 1 and *s*_2_ = −1, corresponding to the standard static values of a stoichiometric matrix. Assume we have *q* = 3 scenarios, and

$${p}_{1}=(\begin{array}{c}\mathrm{1/4}\\ \mathrm{1/2}\\ \mathrm{1/4}\end{array})\quad {\rm{and}}\quad {\hat{S}}_{1}=[\begin{array}{cc}0.9 & -1.2\\ 1 & -1\\ 1.1 & -0.8\end{array}],$$

which means that

$$P({s}_{1}=0.9\,{\rm{and}}\,{s}_{2}=-\mathrm{1.2)}=P({s}_{1}=1.1\,{\rm{and}}\,{s}_{2}=-\mathrm{0.8)}=\mathrm{1/4}$$

and

$$P({s}_{1}=1\,{\rm{and}}\,{s}_{2}=-\mathrm{1)}=\mathrm{1/2}.$$

The expected value of the constraint is simply

$${p}_{1}^{T}{\hat{S}}_{1}v={\bar{S}}_{1}v={v}_{1}-{v}_{2},$$

which is the value of the original, static constraint. Setting *δ*_1−*ε*_ = 3 so that *ε* ≈ 0.0015, we have that

12 $${R}_{1}\approx [\begin{array}{cc}-0.15 & -0.30\\ 0 & 0\\ 0.15 & 0.30\end{array}].$$

If *M*_1_ = 0.2, then Eqs (10) and (11) combine to show that *v*_1_ and *v*_2_ are feasible in the stochastic model if and only if

$$-0.2\le \mathrm{(1}-\mathrm{0.15(}{u}_{1}-{u}_{3})){v}_{1}+(-1-\mathrm{0.30(}{u}_{1}-{u}_{3})){v}_{2}\le 0.2$$

for all vectors *u* such that ${u}_{1}^{2}+{u}_{2}^{2}+{u}_{3}^{2}\le 1$. Hence, *v*_1_ and *v*_2_ are feasible in the stochastic case only if they satisfy an infinite number of linear inequalities that are perturbations of a relaxed version of the original, static equality.

This infinite collection of constraints is neither more nor less restrictive than the FBA constraint. One interpretation is that RAMP first relaxes the average equilibrium constraint of ${p}_{i}^{T}{\hat{S}}_{i}v=0$ by replacing this last expression with $-{M}_{i}\le {p}_{i}^{T}{\hat{S}}_{i}v\le {M}_{i}$, but then RAMP further restricts the relaxed constraint by replacing it with $\Vert {R}_{i}v\Vert -{M}_{i}\le {p}_{i}^{T}{\hat{S}}_{i}v\le {M}_{i}-\Vert {R}_{i}v\Vert$, which adds an infinite number of linear constraints (per original constraint).

**Figure 1:**
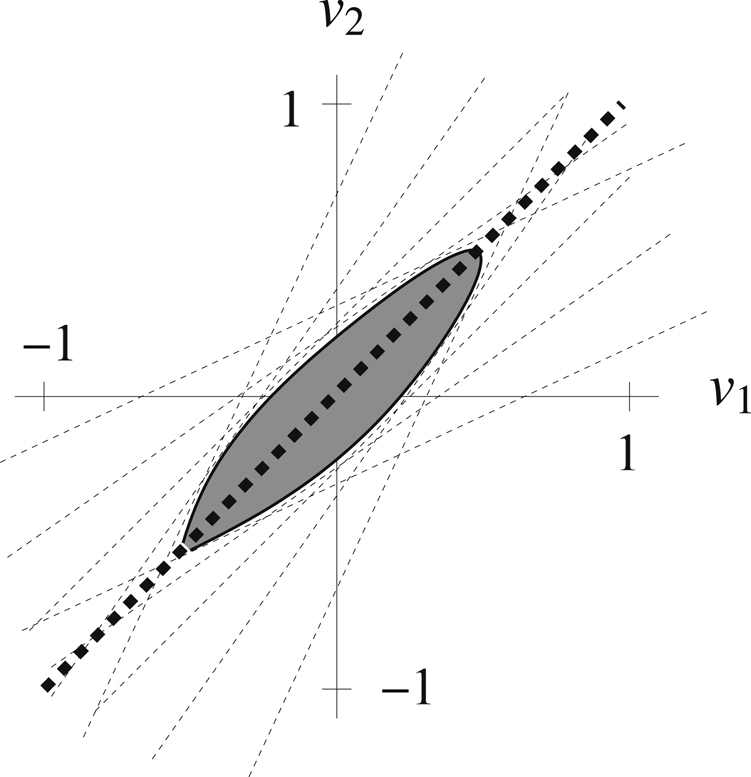
Graphical representation of the RAMP method. A depiction of the difference between an FBA (static) equality (the strong dashed line segment through the origin) and its stochastic RAMP counterpart (the bounded, shaded region). The light dashed lines represent the infinite set of linear constraints added by Eqns (10) and (11).

A depiction of the geometry for the above example is shown in Fig. 1. The original, static (FBA) constraint with the variable bounds is depicted by the bold dashed line segment through the origin. The shaded region is the feasibility set formed by the combined stochastic constraints, and the light dashed lines are samples of the infinite set of linear inequalities added by the stochastic model. The RAMP feasible region does not contain the FBA feasible region. As an example, the point (0.75, 0.75) is feasible to the original static FBA constraint but is infeasible to the stochastic RAMP constraints. Likewise, there are feasible solutions to the stochastic model that are infeasible in the static case.

### Mathematical observations of RAMP properties

In the following we develop three important mathematical qualities of the RAMP model by establishing three Theorems, proofs of which are contained in the Methods section. Our first result demonstrates that feasible RAMP flux vectors satisfy a Lipschitz continuity property in their probabilistic elements, provided that bounding constraints can be marginally relaxed. The second result demonstrates that convergent RAMP solutions are indeed FBA solutions as stochastic uncertainty diminishes. The third theorem characterizes the types of uncertainty in the stoichiometric matrix that can be tolerated by an optimal FBA solution.

#### Continuity property of RAMP solutions

Corollary 1 (see Methods) suggests that RAMP’s SOCP constraints might imbue continuity of the feasible flux vectors with regard to the stochastic modeling elements *p*_*i*_ and ${\hat{S}}_{i}$. However, the linear bounds *L* ≤ *v* ≤ *U* prevent an immediate application of Corollary 1 because realistic genome-scale metabolic models commonly enforce some fluxes with implied equalities, i.e. *L*_*i*_ = *U*_*i*_ for some *i*. An example is the often called ATP maintenance flux, which is the rate at which ATP is lost due to non-growth-associated processes. These fixed fluxes are included to ensure that FBA models account for cellular demands that are not explicitly part of the genome-scale metabolic reconstructed model^39^. The continuity results depend on scaling, and Lemma 1 and Corollary 1 (see Methods) do not provide the Lipschitz continuity for RAMP since scaled fixed fluxes do not remain feasible under the requirement that *v*_*i*_ = *L*_*i*_ = *U*_*i*_.

The equalities imposed by the bounding constraints could be re-cast probabilistically. However, unlike the steady state equations of *Sv* = 0, these constraints cannot be guaranteed to be of the form required by Lemma 1. For instance, if *v*_*i*_ is the flux for the reaction that removes ATP for non-growth-associated demands, then the equality *v*_*i*_ = *L*_*i*_ would naturally transition to *P*(*L*_*i*_ − *M*_*i*_ ≤ *v*_*i*_ ≤ *L*_*i*_ + *M*_*i*_) ≥ 1 − 2*ε*, which would associate with an SOCP constraint of the form ${L}_{i}-{M}_{i}+\Vert {R}_{i}v\Vert \le v\le {L}_{i}+{M}_{i}-\Vert {R}_{i}v\Vert$. Since the signs of *L*_*i*_ − *M*_*i*_ and *L*_*i*_ + *M*_*i*_ would agree for small values of *M*_*i*_, which would be required to remain realistic, the requirement of a positive right-hand side in Lemma 1 can not be ensured after writing both inequalities in the correct form.

Nevertheless, if we adjust the variable bounds dependent on the perturbed stochastic elements instead of prescribing them independent of perturbation, then we can relax the bounds to ensure feasibility. Probabilistically this means that we can set the values of *M*_*i*_ for the variable bounds so that we satisfy them with probability 1.

##### **Theorem 1.**

*For a collection of probability vectors p*_*i*_ *and scenario matrices* ${\hat{S}}_{i}$ *and for the lower and upper bounds U and L*, *let* ${\mathcal F} (\{({p}_{i},{\hat{S}}_{i})\,:i=\mathrm{1,\; 2},\ldots ,m\},L,U)$ *be the nonempty set of feasible fluxes satisfying the constraints of the RAMP model in* Eq. (9). *Assuming that each p*_*i*_ + Δ*p*_*i*_ *is a probability vector*, *we then have for each*

$$v\in  {\mathcal F} (\{({p}_{i},{\hat{S}}_{i})\,:i=\mathrm{1,\; 2},\ldots ,m\},L,U)$$

*that there is a λ* ≥ 0 *such that*

$$\mathop{{\rm{\min }}}\limits_{v^{\prime} }\Vert v-v^{\prime} \Vert \le \lambda {\rm{\Gamma }}$$

*where*

$$\begin{array}{rcl}{\rm{\Gamma }} & = & \mathop{{\rm{\max }}}\limits_{i}\{\Vert {\rm{\Delta }}{p}_{i}\Vert +\Vert {\rm{\Delta }}{\hat{S}}_{i}\Vert +\Vert {\rm{\Delta }}{p}_{i}\Vert \Vert {\rm{\Delta }}{\hat{S}}_{i}\Vert \},\\ v^{\prime}  & \in  &  {\mathcal F} (\{({p}_{i}+{\rm{\Delta }}{p}_{i},{\hat{S}}_{i}+{\rm{\Delta }}{\hat{S}}_{i})\,:i=1,2,\ldots ,m\},L-\lambda {\rm{\Gamma }}e,U+\lambda {\rm{\Gamma }}e),\\  &  & and\,\lambda \,\,is\,independent\,of\,all\,\Vert {\rm{\Delta }}{\hat{S}}_{i}\Vert .\end{array}$$

Theorem 1 highlights that any questionable discontinuities that FBA might exhibit with regard to parametric update are due to the imposed linear equalities that are the byproduct of enforcing the biologically unrealistic assumption of a uniform steady state. The RAMP approach adjusts FBA’s modeling paradigm to include its inherent stochastic nature, and in the process, RAMP ensures the continuity that would be expected of the feasible flux states as long as the imposed linear bounds can also be relaxed commensurate with the magnitude of the perturbation. In conclusion, any discontinuity of FBA that could be caused by minute model adjustments are the outcome of an overly rigid (linear) model.

We note that a recent discussion in the literature, see refs 40–42, suggests that the creation of biomass might be sensitive to small perturbations in the growth coefficients. Indeed, the authors of ref. 40 have argued that in some cases perturbations to the biomass equation are necessary to achieve growth. The continuity of Theorem 1 counters any such concern with regard to RAMP, as flux states for any growth equation would remain close to those with a perturbed equation. Specifically, a change in the creation of biomass is controlled by the amount of coefficient perturbation, and hence, biomass creation can not depend on small adjustments to the growth coefficients in RAMP.

#### Connection between FBA and RAMP solutions

A reasonable question is if the deterministic FBA model is a limiting case of RAMP as the stochastic elements diminish? Theorems 2 and 3 show that convergent RAMP solutions are indeed FBA solutions, and hence, they answer this question in the affirmative. They also show that interpreting FBA as a limiting RAMP model characterizes the possible random flux states among cells in an optimal growth, steady state culture. The convergence result of Theorem 2 assumes an interiority condition, which is tacit as long as the equalities imposed by the bounding constraints are relaxed to allow arbitrarily small adjustments, i.e. as long as *L*_*i*_ = *v*_*i*_ = *U*_*i*_ is replaced with *L*_*i*_ − *η*  ≤  *v*_*i*_ ≤ *U*_*i*_ + *η* for any arbitrarily small *η* > 0. The proof of Theorem 2 (see Methods) is straightforward and follows from the fact that if the primal and dual solutions converge as the stochastic elements dissipate, then the resulting necessary and sufficient conditions are that of FBA. However, the proof importantly identifies that not all random elements need to disappear, a point that prompts Theorem 3.

##### **Theorem 2.**

*Let* ${p}_{i}^{t}$, ${\hat{S}}_{i}^{t}$, ${M}_{i}^{t}$, *be sequences such that for each t the corresponding RAMP model satisfies Slater*’*s interiority condition*. *Let v* ^*t*^ *be an optimal solution of the RAMP model corresponding to* ${p}_{i}^{t}$, ${\hat{S}}_{i}^{t}$, *and* ${M}_{i}^{t}$, *and assume v* ^*t*^ → *v as t* → ∞. *Assume likewise that a corresponding dual sequence of optimal solutions converges*. *Further assume that as t* → ∞ *we have* ${({p}_{i}^{t})}^{T}{\hat{S}}_{i}^{t}\to {\bar{S}}_{i}$, ${R}_{i}^{t}\to 0$, *and* ${M}_{i}^{t}\to 0$. *Then v is a solution to the FBA model*

$${\rm{\max }}\{{v}_{Growth}\,:\bar{S}v=\mathrm{0,}\,L\le v\le M\},$$

*where* $\bar{S}$ *is the matrix whos*e *i*-*th row is* ${\bar{S}}_{i}$.

The assumption that ${R}_{i}^{t}\to 0$ in Theorem 2 can be relaxed since the proof remains valid as long as the limiting matrices *R*_*i*_ satisfy *R*_*i*_ *v* = 0 and ${R}_{i}^{T}({y}_{i}+{w}_{i})=0$ (see the proof in Methods). Since *R*_*i*_ can have low rank, e.g. the rank of *R*_1_ in Eq. (12) is 1, we see that *R*_*i*_ need not generally vanish. This observation suggests that not all uncertainty needs to be removed to recover an FBA solution with RAMP. It is this interesting observation that motivates our third result.

#### Allowed probabilistic variation for optimal flux solution

Suppose $\hat{v}$ is a FBA solution corresponding with an average stoichiometric matrix, i.e. the rows of the matrix are ${p}_{i}^{T}{\hat{S}}_{i}$. We pose the question of whether or not it is possible to identify nontrivial scenarios and probabilities so that $\hat{v}$ is a solution to both an FBA problem and to a limiting stochastic RAMP counterpart. If such probabilities and scenarios exist, then the optimal flux state $\hat{v}$ would be optimal for a range of stochastic variation *within* an optimal growth, steady state culture. We describe scenarios ${\hat{S}}_{i}$ as *biologically possible* for $\hat{v}$ if there exists (positive) probability vectors *p*_*i*_ such that $\hat{v}$ maximizes cellular growth under the conditions that $\hat{v}$ satisfies $L\le \hat{v}\le U$ and

$$\mathop{\mathrm{lim}}\limits_{{M}_{i}\to 0}P(-{M}_{i}\le {p}_{i}^{T}{\hat{S}}_{i}\hat{v}\le {M}_{i})\le 1-2\varepsilon ,\forall i.$$

Theorem 3 characterizes the biologically possible scenarios of any FBA solution. The argument arranges and partitions $\hat{v}$ into $(\hat{v}^{\prime} ,\hat{v}^{\prime\prime} )$, where ${\hat{v}}_{j}^{^{\prime} }\ne 0$ for all *j* and $\hat{v}^{\prime\prime} =0$. This way $S\hat{v}=S^{\prime} \hat{v}^{\prime}$, where *S*′ is the submatrix of *S* whose columns correspond with $\hat{v}^{\prime}$.

##### **Theorem 3.**

*Let* $\hat{v}=(\hat{v}^{\prime} ,\mathrm{0)}$, *with* ${\hat{v}}_{j}^{^{\prime} }\ne 0$ *for all j*, *be a solution to the FBA problem*

13 $${\rm{\max }}\{{v}_{Growth}:{p}_{i}^{T}{\hat{S}}_{i}v=0,\forall i,L\le v\le U\}.$$

*Then the scenarios of* ${\hat{S}}_{i}$ *are biologically possible for* $\hat{v}$ *if and only if for all i we have* ${\hat{S}}_{i}^{^{\prime} }v^{\prime} ={\alpha }_{i}e$ *for some scalar α*_*i*_ ≠ 0.

In biological terms, Theorem 3 identifies the probabilistic variations that are possible in the stoichiometric matrix for cells with the same optimal, steady state fluxes. Thus, if we trust that FBA solutions do indeed identify the limiting behavior of cells as they evolve to optimize to their environmental resources, then Theorem 3 concludes that optimized cells with the same flux state can be described by different probabilistic models.

Additionally, since Eq. (17) in the proof of Theorem 3 only requires that *p*_*i*_ be a probability vector with nonzero entries, we can use this fact to construct a test that will check if any particular collection of scenarios is possible for optimal states. For example, suppose we are interested in investigating if some of the stoichiometric coefficients can vary for the cells having a specified flux state $\hat{v}$ in an optimized culture. Any collection of scenarios for which ${\hat{S}}_{i}\hat{v}$ does not have identical components is impossible, since there is no possible way to assign probabilities that make the flux state optimal once probabilistic variation is included in the model. If our mathematical models accurately assess biological reality, then we can claim that it is biologically impossible to have a collection of optimized cells with a common flux state that vary according to the suggested scenarios.

### RAMP implemented as a computational model

We now compare the computational ability of several RAMP implementations with FBA. The computational results herein only initiate the broad comparisons that would benchmark RAMP against the numerous FBA adaptations. The metrics we use in our benchmarking are direct comparisons between FBA and RAMP predictions of experimentally determined fluxes, and RAMP’s and FBA’s ability to identify essential and viable genes. The latter since this is one of the commonly used tests to assess a metabolic model’s potential for producing biologically relevant predictions. All flux tests were conducted on the iJO1366 metabolic model of *Escherichia coli* ^25^, whereas knockouts also used the iAF1260 metabolic model^24^. These high-quality reconstructed models have, respectively, 1366 and 1260 genes, 2583 and 2382 metabolic reactions, and 1805 and 1668 metabolites.

Gene essentiality is decided by simulating gene knockouts with a corresponding removal of the associated set of metabolic reactions^2, 3^. If the modified metabolic model affects the optimal growth rate, typically by at least a 50% reduction, then the gene is considered to be essential to the organism. When a predictive model correctly identifies a gene as essential, we label this as a true positive. Similarly, a true negative indicates a correctly identified non-essential gene by the predictive model. A false positive occurs if the analysis incorrectly labels a gene as essential, and a false negative if the analysis incorrectly labels a gene as non-essential. The predictive power of the model is the ratio of the sum of true positives and negatives to the number of genes.

The forthcoming computational experiments introduces various stochastic assumptions by querying either different percentiles *δ*_1−*ε*_ or different scenarios for the growth equation. At this point, however, it is necessary to emphasize that the values of *M*_*i*_ bounds are also part of the stochastic interpretation since they define the permissible variation from steady state. Specifically, the set of feasible flux states either grows or stays the same with an increase in any *M*_*i*_, from which we know that the growth rate is non-decreasing as a function of the individual *M*_*i*_ values. So if the *M*_*i*_ values are too small, then we will likely under-approximate the growth rate, but if the *M*_*i*_ values are too large, then we will likely over-approximate the growth rate. We solve an optimization problem (see Methods) to appropriately set the values of *M*_*i*_ so that RAMP’s growth rate exactly matches that of FBA’s. The problem ensures that the sum of permissible variations from steady state is as small as possible.

#### RAMP assessment of uncertainty in growth reaction

The goal of our first computational experiment is to gauge the relationship between RAMP and FBA for individual uncertainties in the growth reaction. This is motivated by the observation that stoichiometric coefficients of the growth reaction are empirically determined non-integers, with values spanning many orders of magnitude (see e.g. Table 1). We address this goal by posing the question, What is the maximum level of uncertainty tolerated in a single stoichiometric coefficient in the growth reaction before RAMP’s predictions of gene-knockout essentiality depart from those of FBA in a given environment?

We tackle this challenge by individually increasing the level of randomness in a single growth coefficient, multiplying it with a factor containing ±*σ* based on five scenarios, and we increase the multiplier *σ* from zero in an iterative fashion (see Methods for details). As expected, RAMP and FBA coincide for the case with no randomness in the growth stoichiometric coefficients, i.e. with *σ* = 0. However, as *σ* increases it is possible that RAMP will find a different set of essential genes than FBA. Consequently, we determine the maximal value of *σ* for which RAMP and FBA return identical sets of essential genes.

Assuming that the genome-scale metabolic reconstruction is a high-quality rendering of the biochemical processes associated with the individual biomass components, we make the following observations: If RAMP returns identical essential gene sets as FBA (i.e. stable predictive ability) for large percent variations of a single coefficient, then the cells of the culture could possibly be disparate in how they incorporate the associated metabolite in biomass. If the predictive ability instead degrades with only slight deviations, then the growth coefficient is more likely exhibiting little variation across the culture.

**Figure 2:**
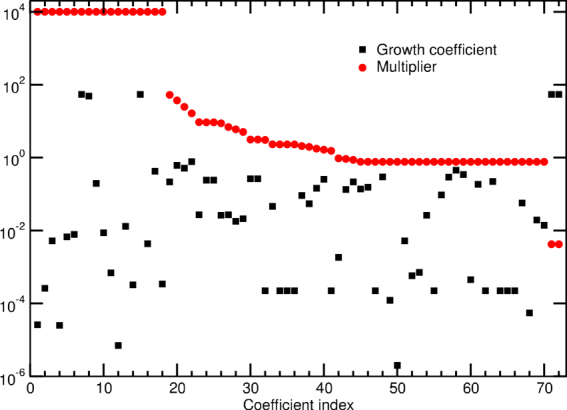
Plot of multipliers and growth coefficients. The largest permissible multiplier *σ* (red) sorted in descending order for each of the 72 growth coefficients in *E*. *coli* model iJO1366^25^. The absolute value of the stoichiometric growth coefficient is shown for comparison (black). Note that the multiplier values for 18 indices (see Table 2 for names) have an undetermined maximal value > 10^26^ and are placed at the vertical axis (10^4^) for completeness.

Figure 2 displays, on a logarithmic scale, the calculated maximal values of the multiplier *σ* before RAMP departs from FBA’s gene-knockout predictions, for each of the 72 growth coefficients in iJO1366^25^. We find that for 18 of the coefficients (see Table 2 for their names), we were only able to determine a lower bound on the value of the multiplier of *σ* > 10^26^. Upon inspection of the numerical results for these 18 coefficients, we noticed the following two properties: within numerical accuracy, (i) the value of the mean (see Eq. (6)) is *μ*_*i*_ = 0, and (ii) the standard deviation (see Eq. (7)) scales with *M*_*i*_. A direct consequence of these two observations is that the RAMP-specific constraints of Eq. (8) reduces to the standard FBA constraint *S*_*i*_ *v* = 0. Hence, for these 18 coefficients of the biomass equation, the RAMP problem reduced to that of FBA.

**Table 2:** Tabulation of model-specific names of the (cytosolic) coefficients in the biomass equation of iJO1366^25^ associated with numerically unbounded multipliers *σ*, thus allowing RAMP to predict gene essentiality identical to that of standard FBA.

2fe2s	4fe4s	cl	cobalt2	fe2	fe3
h	h2o	k	mg2	mn2	mobd
nh4	ni2	pi	so4	val-L	zn2

The biochemical explanation for why RAMP collapses to the standard FBA problem for all values of *σ* is immediate for the coefficients in Table 2 with the exception of 2fe2s, 4fe4s, fe3, and mobd: Upon inspection of iJO1366, we find that the biomass components are (i) freely available in the environment, and (ii) may be imported into the cytosol at no energetic or metabolic burden. Hence, any change of their stoichiometric coefficients in the biomass equation leaves the remainder of the biochemical network flux-patterns unchanged. However, the introduction or generation of 2fe2s, 4fe4s, fe3, and mobd into the cytosolic compartment is associated with metabolic burden through more complex pathways in the biochemical network reconstruction. For these four cases we were unable to determine a clear pattern, making a detailed biochemical explanation difficult.

We find for the remaining 54 biomass coefficients that the maximally allowed multiplier spans a surprising 4 orders of magnitude. Upon investigating a possible relationship between these maximal multiplier values and the corresponding stoichiometric coefficients, we find that they are uncorrelated (*ρ* = −0.004). Note that, for all of the 54 coefficients, the numerically determined RAMP solutions are more permissive than the FBA solutions, meaning that at the determined maximal multiplier values, RAMP identified as essential a subset of the FBA-determined essential genes. In total, we found that 23 of the 54 coefficients supported maximal permissible multipliers *σ* > 1. This is quite surprising, as it suggests that both the sign and the magnitude of the corresponding stoichiometric coefficient may vary while the growth rate and RAMP’s predictive power are maintained.

#### RAMP computational predictions of gene essentiality

The essential gene predictions of RAMP and FBA for our second computational experiment, in which all coefficients in the growth reaction are simultaneously uncertain (see Methods), are tabulated in Table 3. We assumed in all experiments an oxygen-unlimited environment with glucose as the sole, and limiting, carbon source as the default environment. We additionally conducted the same tests with glycerol as the only carbon source, finding results that mirror those of glucose (not shown).

**Table 3:** Predictability of gene knockouts using stochastic models 1, 2, 3, and 4 for RAMP compared with FBA.

	Experimental						
			Essential		Nonessential		
**Computational**	**Essential**	True Positive	FBA	171 (160)	FBA	44 (35)	False Positive
			RAMP_1_	171 (160)	RAMP_1_	44 (35)	
			RAMP_2_	162 (160)	RAMP_2_	43 (35)	
			RAMP_3*a*_	166 (155)	RAMP_3*a*_	44 (35)	
			RAMP_3*b*_	166 (152)	RAMP_3*b*_	44 (30)	
			RAMP_3*c*_	163 (152)	RAMP_3*c*_	39 (30)	
			RAMP_4_	171 (160)	RAMP_4_	44 (35)	
	**Nonessential**	False Negative	FBA	77 (78)	FBA	1074 (987)	True Negative
			RAMP_1_	77 (78)	RAMP_1_	1074 (987)	
			RAMP_2_	86 (78)	RAMP_2_	1075 (987)	
			RAMP_3*a*_	82 (83)	RAMP_3*a*_	1074 (987)	
			RAMP_3*b*_	82 (86)	RAMP_3*b*_	1074 (992)	
			RAMP_3*c*_	85 (86)	RAMP_3*c*_	1079 (992)	
			RAMP_4_	77 (78)	RAMP_4_	1074 (987)	

RAMP models 3a, 3b, and 3c have increasing levels of stochastic variation, respectively. Results for the iAF1260 metabolic model are in parentheses, all other results are for the iJO1366 model.

The tallies in Table 3 show some interesting differences between the two genome-scale metabolic reconstructions iAF1260^24^ and iJO1366^25^ in response to the different RAMP implementations. While the majority of biochemical reactions are the same in these two models, the RAMP analysis emphasizes that small details of the reconstructed model affect the identification of essential genes. Here, RAMP may be an additional approach in vetting the quality of model reconstructions.

Table 3 shows that the default RAMP model (RAMP_1_), which only permits uncertainty past the stated accuracy of FBA’s growth equation, precisely replicates the essential gene predictions of FBA for both genome-scale metabolic model reconstructions. This fact computationally substantiates Theorem 2 and numerically demonstrates further that minor probabilistic variations mimic FBA.

Model 2 (RAMP_2_) is designed to magnify the perturbations of Model 1 by a multiplicative factor *ρ* and ‘spread’ the scenarios in the last few digits. From Table 3, we see that this approach impacts RAMP’s predictive ability. For the iJO1366 model, all values of *ρ* decreased the true positives by 9 and increased the true negatives by 1, which means that RAMP_2_ had 8 fewer correct predictions than FBA with increased scenario variability as magnified by *ρ*. In contrast, the iAF1260 model agreed with FBA for all values of *ρ*. RAMP’s predictive power compared with FBA for RAMP_2_ decreased from 91.14% to 90.56% with the iJO1366 model and matched FBA’s 91.32% with the iAF1260 model. The similarity between FBA and RAMP for RAMP_1_ and RAMP_2_ shows that probabilistic deviations in the growth coefficients beyond what is reported by the genome-scale metabolic reconstructions only lead to minor adjustments in predictive power.

Scaling the scenarios proportionately (RAMP_3*a*_, RAMP_3*b*_, and RAMP_3*c*_ representing increasing levels of variation) has a slightly more varied effect than multiplying the default variations by *ρ*. Results with *σ* ≥ 0.4 had little value since these models relaxed the FBA constraints to the point at which few, if any, essential genes were identified, and for this reason we only report the results for *σ* ≤ 0.3. The trend as *σ* increases is that RAMP_3_ reduces the number of predicted essential genes, which lowers the number of true and false positives but increases the number of true and false negatives. Five true positives move to false negatives in the iJO1366 model for RAMP_3*a*_ and RAMP_3*b*_, which reduces RAMP’s predictive ability to 90.78%. However, for larger *σ*, RAMP_3*c*_ increases its number of true negatives and reduces the number of false positives, which increased predictive ability to 90.92%. The trend for the iAF1260 model is similar.

Altering the parameter *ε* (RAMP_4_) had no effect on RAMP’s ability to predict essential genes, and for all tested values of *ε*, RAMP agreed with FBA. This clearly suggests that it is more important to tune RAMP with regard to the scenarios than it is with the probabilistic guarantee of satisfying the near equilibrium constraints in Eq. (3).

#### RAMP comparison with experimentally determined fluxes

**Figure 3:**
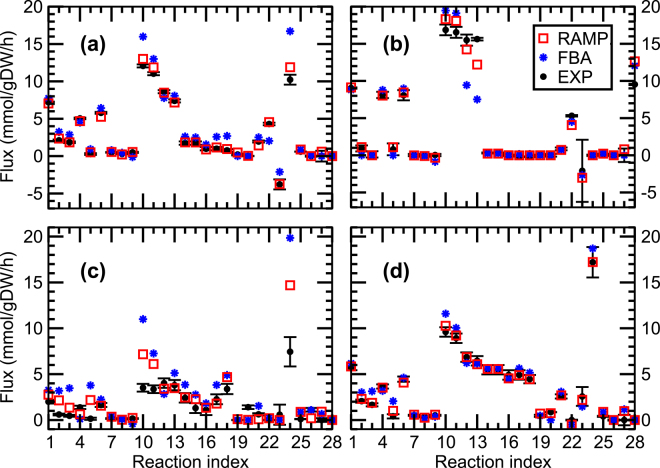
Comparison of experimental fluxes with RAMP and FBA predictions. Experimentally determined fluxes in *E*. *coli* (black circles with whiskers) for 28 reactions in the central carbon metabolism are compared with the predictions from FBA (blue stars) and from RAMP_3_ with *σ* = 0.2 (red squares). All calculations are made with model iJO1366^25^. The panels correspond to experiments conducted under conditions of (**a**) aerobic batch growth^26^, (**b**) anaerobic batch growth^26^, (**c**) carbon-limited chemostat at dilution rate 0.1/h^27^, and (**d**) carbon-limited chemostat at dilution rate 0.4/h^27^. The correspondence between reaction index and biochemical reaction is given in Supplementary Table S1.

To investigate RAMP’s consistency with experimentally determined fluxes, we formulated a non-linear (quadratic) objective function for RAMP that minimizes the distance between a set of experimentally determined flux data and the corresponding reaction fluxes in a genome-scale metabolic model. We directly compare the results from RAMP_3_ with *σ* = 0.2 with the corresponding results from FBA. The details of the problem formulation are given in Eqs (22) and (23). We used flux data^6^ measured for 28 reactions from the central carbon metabolism of *E*. *coli* growing on glucose in batch conditions^26^ both aerobically (Fig. 3(a)) and anaerobically (Fig. 3(b)), as well as in aerobic chemostat conditions^27^ at the two dilution rates of 0.1/h (Fig. 3(c)) and 0.4/h (Fig. 3d)).

A striking visual feature of Fig. 3 is that the RAMP-predicted fluxes consistently provide a better fit to the experimental fluxes than the FBA-predicted ones. In Table 4, we quantify the performance of RAMP and FBA in predicting fluxes by calculating the mean square error ${\rm{MSE}}={\Vert {v}_{predicted}-{v}_{exp}\Vert }^{2}/N$, with *N* = 28. The results reported in Fig. 3 and Table 4 demonstrate that RAMP significantly outperforms FBA in predicting experimental fluxes in a wide range of conditions.

**Table 4:** Quantification of RAMP and FBA ability to predict experimental fluxes.

	RAMP MSE	Relative MSE
Fig. 3(a)	0.171	5.8%
Fig. 3(b)	1.105	24.4%
Fig. 3(c)	3.116	31.7%
Fig. 3(d)	0.106	15.1%

Mean square error (MSE, see text for definition) for RAMP, and relative MSE: the RAMP MSE divided by the FBA MSE.

## Discussion

The expanding suite of systems-biology computational methods described as constraint-based analyses has significantly improved our ability to probe the properties of genome-scale metabolic reconstructions. However, the basic premises of these methods, the assumptions of steady state and no heterogeneity in a cellular population, has stayed unchanged. In this article, we have developed the conceptual framework for a new paradigm of constraint-based methods by explicitly relaxing the basic assumption of steady-state while allowing for heterogeneity. We have presented the mathematical foundation proving that, as levels of uncertainty are reduced, the RAMP method will become identical to standard FBA.

Building on the RAMP foundation, it will be possible to develop robust versions of many of the contemporary constraint-based methods. For some of the new extensions to FBA, the inclusion of RAMP formalism is straightforward, e.g. FBAwMC^32^, GIMME^33^, MOMENT^34^, and GX-FBA^35^. However, also methods that add gene-regulatory effects, such as rFBA^43^, and thermodynamic considerations^44^ may be directly implemented with RAMP, since many of these methods generate modified metabolic networks, or temporal sequences of such. Note that, since it is known that uncertainty in variable bounds, e.g. restrictions such as upper bounds on reaction fluxes, mathematically is not different from uncertainty in the stoichiometric coefficients^20^, the RAMP formulation is also capable of modelling uncertainty in flux constraints.

Theoretically, we are able to formulate a wide host of linear and nonlinear RAMP models as SOCPs. However, the nonlinear ones have not been uniformly translatable into computational reality. While we have been able to solve the *L*_2_ RAMP flux-minimization problem (see Eq. (22)) and show that RAMP outperforms FBA for flux predictions, we have achieved interspersed success with native SOCP solvers for general RAMP models. Specifically, we have attempted to

- solve *L*_2_ versions of Eq. (21),
- predict essential genes with a RAMP version of the minimization of metabolic adjustment (MOMA) method^45^, which is a quadratic model, and
- increase variation beyond the growth coefficients to replicate stochastic environmental bounds.

While we are able to solve these problems occasionally, we were unable to identify a choice of solver settings and convergence criteria to achieve consistent and stable success for the numerical implementation with current SOCP software for any of these extensions. Establishing a stable computational platform for the general RAMP paradigm is an important goal for future work.

The SOCP constraints of RAMP appropriately inject statistical variation into the FBA paradigm, but the conclusion of whether or not RAMP’s stochastic perspective is more or less restrictive than FBA’s deterministic setting remains unclear. From a mathematical point of view, RAMP is neither a direct relaxation of FBA nor a direct restriction of FBA. However, our computational results demonstrate that RAMP is, in practice, a relaxation of FBA. When investigating uncertainty in individual growth coefficients, we uniformly show that discrepancies in gene essentiality are due to RAMP being less restrictive, as evidenced by RAMP having predicted at least one essential gene less than FBA, i.e. a subset of the FBA results. Similarly with all growth coefficients assumed uncertain, RAMP’s stochastic adaptation results in a model whose trend is to predict fewer essential genes, especially as uncertainty increases.

These computational outcomes favorably agrees with the biological sentiment that cellular metabolic networks are more robust in their wild-type setting and are increasingly fragile as they evolve toward an optimal, and specialized, steady-state. Hence, the number of essential genes should reduce as statistical variation increases, which is predicted by RAMP. Moreover, our mathematical analysis and computational outcomes show that RAMP mimics FBA if the stochastic elements are sufficiently small. In particular, if variation is not allowed beyond the assumed accuracy of an FBA model, then RAMP behaves like FBA for predictions of essential genes.

Theorem 3 provides a new approach to discriminate FBA’s optimal flux states: For example, some FBA solutions may have no biologically possible scenarios while others may have many. This suggests that the former is less stable with regards to random variation, whereas the latter is optimal under a wider range of stochastic possibilities. One may intuit that actual flux states should be robust against random variation^46^, and hence, learning to calculate FBA solutions that can witness as much variability as possible is a promising direction of future research.

Finally, our choice of letting the objective function coincide with the growth reaction was purely a choice based on making RAMP easily comparable to traditional implementations of FBA, and RAMP is valid beyond this choice.

## Methods

In this section we provide the mathematical foundation and proofs of the theorems presented in the main text. We also discuss aspects of the computational stability and implementation of RAMP.

**Lemma 1**

The following lemma establishes a general property from which our result follows.

### Lemma 1

*Let* $\bar{A}$ *be an n*-*element row vector*, *R be a q* × *n matrix*, *and b be a positive scalar*. *Let* ${\mathcal F} (\bar{A},R)=\{v\,:\bar{A}v+\Vert Rv\Vert \le b\}$. *Then*, *for any* $v\in F(\bar{A},R)$, *there are scalars z and λ satisfying* 0 < *z* ≤ 1 *and λ* ≥ 0 *such that*

$${\rm{\min }}\{\Vert v-v^{\prime} \Vert \,:v^{\prime} \in  {\mathcal F} (\bar{A}+{\rm{\Delta }}\bar{A},R+{\rm{\Delta }}R)\}\le \Vert v-zv\Vert \le \lambda (\Vert {\rm{\Delta }}\bar{A}\Vert +\Vert {\rm{\Delta }}R\Vert ),$$

*where* $zv\in  {\mathcal F} (\bar{A}+{\rm{\Delta }}\bar{A},R+{\rm{\Delta }}R)$ *and λ is independent of* $\Vert {\rm{\Delta }}\bar{A}\Vert$ *and* $\Vert {\rm{\Delta }}R\Vert$.

### *Proof*

Should *v* satisfy $(\bar{A}+{\rm{\Delta }}\bar{A})v+\Vert (R+{\rm{\Delta }}R)v\Vert \le b$, then we immediately have

$${\rm{\min }}\,\{\Vert v-v^{\prime} \Vert \,:v^{\prime} \in  {\mathcal F} (\bar{A}+{\rm{\Delta }}\bar{A},R+{\rm{\Delta }}R)\}=\Vert v-v\Vert =\mathrm{0,}$$

and we are done with *z* = 1 and *λ* = 0.

Suppose instead that $(\bar{A}+{\rm{\Delta }}\bar{A})v+\Vert (R+{\rm{\Delta }}R)v\Vert  > b$. From the Intermediate Value Theorem there is a *τ* such that 0 < *τ* ≤ 1 and

$$0 < (\bar{A}+\mathrm{(1}-\tau ){\rm{\Delta }}\bar{A})v+\Vert (R+\mathrm{(1}-\tau ){\rm{\Delta }}R)v\Vert =b.$$

To ease notation, let $\bar{A}^{\prime} =\bar{A}+\mathrm{(1}-\tau ){\rm{\Delta }}\bar{A}$ and *R*′ = *R* + (1 − *τ*)Δ*R*. Also select *z* such that

$$z=\frac{b}{b+\tau (\Vert {\rm{\Delta }}\bar{A}\Vert +\Vert {\rm{\Delta }}R\Vert )\Vert v\Vert }.$$

Then,

$$\begin{array}{rcl}(\bar{A}+{\rm{\Delta }}\bar{A})(zv)+\Vert (R+{\rm{\Delta }}R)(zv)\Vert -b & = & (\bar{A}^{\prime} +\tau {\rm{\Delta }}\bar{A})(zv)+\Vert (R^{\prime} +\tau {\rm{\Delta }}R)(zv)\Vert -b\\  & \le  & z(\bar{A}^{\prime} v+\Vert R^{\prime} v\Vert +\tau (\Vert {\rm{\Delta }}\bar{A}\Vert +\Vert {\rm{\Delta }}R\Vert )\Vert v\Vert )-b\\  & = & z(b+\tau (\Vert {\rm{\Delta }}\bar{A}\Vert +\Vert {\rm{\Delta }}R\Vert )\Vert v\Vert )-b=0.\end{array}$$

We conclude that $(\bar{A}+{\rm{\Delta }}\bar{A})(zv)+\Vert (R+{\rm{\Delta }}R)(zv)\Vert \le b$, and hence,

$$zv\in  {\mathcal F} (\bar{A}+{\rm{\Delta }}\bar{A},R+{\rm{\Delta }}R\mathrm{)}.$$

Let $\lambda ={\Vert v\Vert }^{2}/b$, from which we have that

$$\begin{array}{rcl}\Vert v-zv\Vert  & = & (1-\frac{b}{b+\tau (\Vert {\rm{\Delta }}\bar{A}\Vert +\Vert {\rm{\Delta }}R\Vert )\Vert v\Vert })\Vert v\Vert \\  & = & (\frac{\tau {\Vert v\Vert }^{2}}{b+\tau (\Vert {\rm{\Delta }}\bar{A}\Vert +\Vert {\rm{\Delta }}R\Vert )\Vert v\Vert })(\Vert {\rm{\Delta }}\bar{A}\Vert +\Vert {\rm{\Delta }}R\Vert )\\  & \le  & (\frac{{\Vert v\Vert }^{2}}{b})(\Vert {\rm{\Delta }}\bar{A}\Vert +\Vert {\rm{\Delta }}R\Vert )\\  & = & \lambda (\Vert {\rm{\Delta }}\bar{A}\Vert +\Vert {\rm{\Delta }}R\Vert ).\end{array}$$

Hence,

$${\rm{\min }}\{\Vert v-v^{\prime} \Vert \,:v^{\prime} \in  {\mathcal F} (\bar{A}+{\rm{\Delta }}\bar{A},R+{\rm{\Delta }}R)\}\le \Vert v-zv\Vert \le \lambda (\Vert {\rm{\Delta }}\bar{A}\Vert +\Vert {\rm{\Delta }}R\Vert ),$$

and *λ* is independent of $\Vert {\rm{\Delta }}\bar{A}\Vert$ and $\Vert {\rm{\Delta }}R\Vert$.□

Lemma 1 shows that vectors satisfying SOCP constraints of the form $\bar{A}v+\Vert Rv\Vert \le b$, with *b* > 0, can be scaled to remain feasible, and moreover, that the magnitude of the adjustment to remain feasible is uniformly bounded by the magnitude of the perturbations. Importantly, this result immediately extends to finite collections of SOCP constraints of the same form, a fact we formalize in Corollary 1.

### Corollary 1

*For i* = 1, …, *m*, *let* ${\bar{A}}_{i}$ *be an n*-*element row vector*, *R*_*i*_ *be a q* × *n matrix*, *and b*_*i*_ *be a positive scalar*. *Let*

$$ {\mathcal F} (\{({\bar{A}}_{i},{R}_{i})\,:i=1,2,\ldots ,m\})=\{v\,:{\bar{A}}_{i}v+\Vert {R}_{i}v\Vert \le {b}_{i},for\,i=1,2,\ldots m,\}.$$

*Then*, *for any* $v\in  {\mathcal F} (\{({\bar{A}}_{i},{R}_{i})\,:i=1,2,\ldots ,m\})$, *there are scalars z and λ satisfying* 0 < *z* ≤ 1 *and λ* ≥ 0 *such that*

$$\begin{array}{lll}{\rm{\min }}\{\Vert v-v^{\prime} \Vert \,:v^{\prime}  & \in  &  {\mathcal F} (\{{\bar{A}}_{i}+{\rm{\Delta }}{\bar{A}}_{i},{R}_{i}+{\rm{\Delta }}{R}_{i},i=1,2,\ldots ,m\})\}\\  & \le  & \Vert v-zv\Vert \le \lambda \,{{\rm{\max }}}_{i}\{(\Vert {\rm{\Delta }}{\bar{A}}_{i}\Vert +\Vert {\rm{\Delta }}{R}_{i}\Vert )\},\end{array}$$

*where* $zv\in  {\mathcal F} (\{({\bar{A}}_{i}+{\rm{\Delta }}{\bar{A}}_{i},{R}_{i}+{\rm{\Delta }}{R}_{i})\,:i=1,2,\ldots ,m\})$ *and λ is independent of all* $\Vert {\rm{\Delta }}{\bar{A}}_{i}\Vert$ *and* $\Vert {\rm{\Delta }}{R}_{i}\Vert$.

### *Proof*

The proof follows directly from Lemma 1 and its proof upon letting *z*_*i*_ and *λ*_*I*_ be the scalars for each *I* and setting *z* = min_*i*_{*z*_*i*_} and *λ* = max_*i*_{*λ*_*i*_}.□

Unfortunately, systems of linear equalities like those of FBA do not satisfy similar continuity properties, and changing the stoichiometric matrix in FBA can lead to discontinuities. As a simple example, the system of homogeneous equations

$$\begin{array}{rcl}{v}_{1}-{v}_{2} & = & 0\\ {v}_{1}-\mathrm{(1}+\alpha ){v}_{2} & = & 0\end{array}$$

has substantially different solution sets around *α* = 0. Notice that *v*_1_ = *v*_2_ = *t* solves the system for any *t* if *α* = 0, but that the only solution for *α* ≠ 0 is *v*_1_ = *v*_2_ = 0. Hence the solution *v*_1_ = *v*_2_ = 1 at *α* = 0 is not arbitrarily close to a solution with *α* ≠ 0.

**Proof for Theorem 1**

### *Proof*

Let ${\bar{A}}_{i}={p}_{i}^{T}{\hat{S}}_{i}$ and ${\bar{A}}_{i}+{\rm{\Delta }}{\bar{A}}_{i}={({p}_{i}+{\rm{\Delta }}{p}_{i})}^{T}({\hat{S}}_{i}+{\rm{\Delta }}{\hat{S}}_{i})$. Then,

14 $$\begin{array}{rcl}\Vert {\rm{\Delta }}{\bar{A}}_{i}\Vert  & = & \Vert ({p}_{i}^{T}+{\rm{\Delta }}{p}_{i}^{T})({\hat{S}}_{i}+{\rm{\Delta }}{\hat{S}}_{i})v-{p}_{i}^{T}{\hat{S}}_{i}v\Vert \le \Vert {p}_{i}^{T}{\rm{\Delta }}{\hat{S}}_{i}+{\rm{\Delta }}{p}_{i}^{T}{\hat{S}}_{i}+{\rm{\Delta }}{p}_{i}^{T}{\rm{\Delta }}{\hat{S}}_{i}\Vert \Vert v\Vert \\  & \le  & {\kappa }_{i}^{A}(\Vert {\rm{\Delta }}{\hat{S}}_{i}\Vert +\Vert {\rm{\Delta }}{p}_{i}\Vert +\Vert {\rm{\Delta }}{\hat{S}}_{i}\Vert \Vert {\rm{\Delta }}{p}_{i}\Vert ),\end{array}$$

where ${\kappa }_{i}^{A}=\Vert v\Vert {\rm{\max }}\{\Vert {\hat{S}}_{i}\Vert ,\Vert {p}_{i}\Vert ,1\}$. Further note that

15 $$\begin{array}{rcl}\Vert {\rm{\Delta }}{R}_{i}\Vert  & = & \Vert ({R}_{i}+{\rm{\Delta }}{R}_{i})-{R}_{i}\Vert \\  & = & {\delta }_{1-\varepsilon }\Vert \sqrt{{P}_{i}+{\rm{\Delta }}{P}_{i}}(I-e{({p}_{i}+{\rm{\Delta }}{p}_{i})}^{T})({\hat{S}}_{i}+{\rm{\Delta }}{\hat{S}}_{i})-\sqrt{{P}_{i}}(I-e{p}_{i}^{T}){\hat{S}}_{i}\\  & = & {\delta }_{1-\varepsilon }\Vert (\sqrt{{P}_{i}+{\rm{\Delta }}{P}_{i}}-\sqrt{{P}_{i}})(I-e{p}_{i}^{T}){\hat{S}}_{i}+\sqrt{{P}_{i}+{\rm{\Delta }}{P}_{i}}((I-e{p}_{i}^{T}){\rm{\Delta }}{\hat{S}}_{i}-e{\rm{\Delta }}{p}_{i}^{T}{\hat{S}}_{i}-e{\rm{\Delta }}{p}_{i}^{T}{\rm{\Delta }}{\hat{S}}_{i})\Vert \\  & \le  & {\delta }_{1-\varepsilon }(\Vert \sqrt{{P}_{i}+{\rm{\Delta }}{P}_{i}}-\sqrt{{P}_{i}}\Vert \Vert I-e{p}_{i}^{T}\Vert \Vert {\hat{S}}_{i}\Vert +\Vert I-e{p}_{i}^{T}\Vert \times \Vert {\rm{\Delta }}{\hat{S}}_{i}\Vert +\sqrt{q}\Vert {\rm{\Delta }}{p}_{i}\Vert \Vert {\hat{S}}_{i}\Vert +\sqrt{q}\Vert {\rm{\Delta }}{p}_{i}\Vert \Vert {\rm{\Delta }}{\hat{S}}_{i}\Vert ).\end{array}$$

From the Mean Value Theorem there is a *μ*_*ik*_ between *p*_*ik*_ and *p*_*ik*_ + Δ*p*_*ik*_ such that

$$\sqrt{{p}_{ik}+{\rm{\Delta }}{p}_{ik}}-\sqrt{{p}_{ik}}=\frac{{\rm{\Delta }}{p}_{ik}}{2\sqrt{{\mu }_{ik}}}.$$

Hence,

$$\Vert \sqrt{{P}_{i}+{\rm{\Delta }}{P}_{i}}-\sqrt{{P}_{i}}\Vert =\sqrt{\sum _{k=1}^{q}{(\sqrt{{p}_{ik}+{\rm{\Delta }}{p}_{ik}}-\sqrt{{p}_{ik}})}^{2}}=\sqrt{{\sum _{k=1}^{q}(\frac{{\rm{\Delta }}{p}_{ik}}{2\sqrt{{\mu }_{ik}}})}^{2}}\le \frac{1}{2\sqrt{{\mu }_{i}}}\Vert {\rm{\Delta }}{p}_{i}\Vert ,$$

where ${\mu }_{i}={{\rm{\min }}}_{k}\{{\mu }_{ik}\}$. We now have from inequality Eq. (15) that

16 $$\Vert {\rm{\Delta }}{R}_{i}\Vert \le {\kappa }_{i}^{R}(\Vert {\rm{\Delta }}{\hat{S}}_{i}\Vert +\Vert {\rm{\Delta }}{p}_{i}\Vert +\Vert {\rm{\Delta }}{\hat{S}}_{i}\Vert \Vert {\rm{\Delta }}{p}_{i}\Vert ),$$

where

$${\kappa }_{i}^{R}={\delta }_{1-\varepsilon }\,{\rm{\max }}\{\frac{1}{2\sqrt{{\mu }_{i}}}(\Vert I-e{p}_{i}^{T}\Vert +\sqrt{q})\Vert {\hat{S}}_{i}\Vert ,\Vert I-e{p}_{i}^{T}\Vert ,\sqrt{q}\}.$$

Each of RAMP’s SOCP constraints may be re-written as

$$\begin{array}{rcl}({\bar{A}}_{i}+{\rm{\Delta }}{\bar{A}}_{i})v+\Vert ({R}_{i}+{\rm{\Delta }}{R}_{i})v\Vert  & \le  & {M}_{i},i=1,2,\ldots ,{\rm{m}}\,\,{\rm{and}}\\ -({\bar{A}}_{i}+{\rm{\Delta }}{\bar{A}}_{i})v+\Vert ({R}_{i}+{\rm{\Delta }}{R}_{i})v\Vert  & \le  & {M}_{i},i=1,2,\ldots ,m.\end{array}$$

From Corollary 1 and inequalities Eqs (14) and (16) we know that there is a *v*′ satisfying these perturbed SOCP constraints and a $\hat{\lambda }\ge 0$ defined independent of $\Vert {\rm{\Delta }}{A}_{i}\Vert$ and $\Vert {\rm{\Delta }}{R}_{i}\Vert$, and subsequently independent of $\Vert {\rm{\Delta }}{\hat{S}}_{i}\Vert$, such that

$$\begin{array}{rcl}\Vert v-v^{\prime} \Vert  & \le  & \hat{\lambda }\mathop{{\rm{\max }}}\limits_{i}\{\Vert {\rm{\Delta }}{\bar{A}}_{i}\Vert +\Vert {\rm{\Delta }}{R}_{i}\Vert \}\\  & \le  & \hat{\lambda }\mathop{{\rm{\max }}}\limits_{i}\{({\kappa }_{i}^{A}+{\kappa }_{i}^{R})(\Vert {\rm{\Delta }}{\hat{S}}_{i}\Vert +\Vert {\rm{\Delta }}{p}_{i}\Vert +\Vert {\rm{\Delta }}{\hat{S}}_{i}\Vert \Vert {\rm{\Delta }}{p}_{i}\Vert )\}\\  & \le  & \hat{\lambda }\mathop{{\rm{\max }}}\limits_{i}\{{\kappa }_{i}^{A}+{\kappa }_{i}^{R}\}\mathop{{\rm{\max }}}\limits_{i}\{(\Vert {\rm{\Delta }}{\hat{S}}_{i}\Vert +\Vert {\rm{\Delta }}{p}_{i}\Vert +\Vert {\rm{\Delta }}{\hat{S}}_{i}\Vert \Vert {\rm{\Delta }}{p}_{i}\Vert )\}.\end{array}$$

Since $\hat{\lambda }$, ${\kappa }_{i}^{A}$, and ${\kappa }_{i}^{R}$ are all independent of $\Vert {\rm{\Delta }}{\hat{S}}_{i}\Vert$, the proof is complete upon noticing that *v*′ must also satisfy

$$L-\lambda \,{\rm{\Gamma }}e\le v^{\prime} \le U+\lambda {\rm{\Gamma }}e,$$

where $$\lambda =\hat{\lambda }{{\rm{\max }}}_{i}\{{\kappa }_{i}^{A}+{\kappa }_{i}^{R}\}$$ and

$${\rm{\Gamma }}=\mathop{{\rm{\max }}}\limits_{i}\{\Vert {\rm{\Delta }}{p}_{i}\Vert +\Vert {\rm{\Delta }}{\hat{S}}_{i}\Vert +\Vert {\rm{\Delta }}{p}_{i}\Vert \Vert {\rm{\Delta }}{\hat{S}}_{i}\Vert \}.$$

**Proof for Theorem 2**

### *Proof*

For each *t* let $({\hat{y}}^{t},{y}^{t},{\hat{w}}^{t},{w}^{t},{\rho }^{t},{\sigma }^{t})$ be the assumed dual solution that converges to $(\hat{y},y,\hat{w},w,\rho ,\sigma )$. Further let *f* be a vector so that *f* ^*T*^ *v* = *v*_Growth_. The dual can be assumed to satisfy Slater’s interiority condition since the necessary and sufficient primal-dual conditions in this case would be satisfied by the convergent sequences:

$$\begin{array}{c}-{M}_{i}^{t}+\Vert {R}_{i}^{t}{v}^{t}\Vert \le {({p}_{i}^{t})}^{T}{\hat{S}}_{i}^{t}{v}^{t}\le {M}_{i}^{t}-\Vert {R}_{i}^{t}{v}^{t}\Vert ,\forall i\\ L\le {v}^{t}\le U\\ \sum _{i}({({\hat{S}}_{i}^{t})}^{T}{p}_{i}^{t}({\hat{y}}_{i}^{t}-{\hat{w}}_{i}^{t})-{({R}_{i}^{t})}^{T}({y}_{i}+{w}_{i}))+{\rho }^{t}-{\sigma }^{t}=f\\ \Vert {y}_{i}^{t}\Vert \le {\hat{y}}_{i}^{t},\forall i\\ \Vert {w}_{i}^{t}\Vert \le {\hat{w}}_{i}^{t},\forall i\\ {\rho }^{t},{\sigma }^{t}\ge 0\\ \sum _{i}{M}_{i}^{t}({\hat{y}}_{i}^{t}+{\hat{w}}_{i}^{t})+{U}^{T}{\rho }^{t}-{L}^{T}{\sigma }^{t}-{f}^{T}{v}^{t}=0.\end{array}$$

Dual feasibility is defined by the 3^rd^ through 6^th^ constraints, from which we can see that the dual is always strictly feasible, and hence, the dual satisfies Slater’s interiority condition. Select any *y* and *w* variables such that $\Vert {y}_{i}^{t}\Vert  < {\hat{y}}_{i}^{t}$ and $\Vert {w}_{i}\Vert  < {\hat{w}}_{i}^{t}$. Since *ρ* ^*t*^ − *σ* ^*t*^ can attain any vector, these two variables can be chosen so that *ρ* ^*t*^ > 0, *σ* ^*t*^ > 0 and that

$$\sum _{i}({({\hat{S}}_{i}^{t})}^{T}{p}_{i}^{t}({\hat{y}}_{i}^{t}-{\hat{w}}_{i}^{t})-{({R}_{i}^{t})}^{T}({y}_{i}^{t}+{w}_{i}^{t}))+{\rho }^{t}-{\sigma }^{t}=f.$$

Hence the system is indeed necessary and sufficient for optimality.

Allowing *t* → ∞, we have by assumption that ${({p}_{i}^{t})}^{T}{\hat{S}}_{i}^{t}\to {\bar{S}}_{i}$, ${R}_{i}^{t}\to 0$, and ${M}_{i}^{t}\to 0$, from which we have

$$\begin{array}{c}\bar{S}v=0\\ L\le v\le U\\ {\bar{S}}^{T}(\hat{y}-\hat{w})+\rho -\sigma =f\\ \hat{y},\hat{w},\rho ,\sigma \ge 0\\ {U}^{T}\rho -{L}^{T}\sigma -{f}^{T}v=0.\end{array}$$

Since these are the necessary and sufficient conditions for FBA, *v* is an optimal solution to the FBA model.

**Proof for Theorem 3**

### *Proof*

Let $\hat{v}=(v^{\prime} ,0)$ be as stated. Then,

17 $${R}_{i}\hat{v}={R}_{i}^{^{\prime} }\hat{v}^{\prime} =\sqrt{{P}_{i}}(I-e{p}_{i}^{T})\hat{S}^{\prime} \hat{v}^{\prime} =0\quad {\rm{if}}\,{\rm{and}}\,{\rm{only}}\,{\rm{if}}\quad e{p}_{i}^{T}(\hat{S}^{\prime} \hat{v}^{\prime} )=(\hat{S}^{\prime} \hat{v}^{\prime} ).$$

The last equality shows that ${R}_{i}\hat{v}=0$ if and only if $\hat{S}^{\prime} \hat{v}^{\prime}$ is an eigenvector of $e{p}_{i}^{T}$ for the eigenvalue 1. Since *p*_*i*_ is a probability vector, $e{p}_{i}^{T}$ has only two eigenspaces, one of dimension *q* − 1 for the eigenvalue 0, and one of dimension 1 for the eigenvalue 1. All eigenvectors for the eigenvalue of 1 are scalar multiples of the all ones vector *e*. Hence, ${R}_{i}\hat{v}=0$ if and only if the scenarios for each *I* satisfy ${\hat{S}}_{i}^{^{\prime} }{\hat{v}}_{i}^{^{\prime} }={\alpha }_{i}e$ for some *α*_*I*_ ≠ 0.

RAMP’s SOCP constraints as *M*_*i*_ ↓ 0 are

$$\Vert {R}_{i}v\Vert \le {p}_{i}^{T}{\hat{S}}_{i}v\le -\Vert {R}_{i}v\Vert \iff \{\begin{array}{c}{p}_{i}^{T}{\hat{S}}_{i}v=0\\ {R}_{i}v=0.\end{array}$$

Hence, the limiting RAMP model as *M*_*i*_ ↓ 0 is the linear program

18 $${\rm{\max }}\{{v}_{{\rm{Growth}}}\,:{p}_{i}^{T}{\hat{S}}_{i}v=0,{R}_{i}v=\mathrm{0,}\forall i,L\le v\le U\}.$$

The necessary and sufficient conditions are

19 $$\begin{array}{c}{p}_{i}{\hat{S}}_{i}v=0,\forall i\\ {R}_{i}v=0,\forall i\\ L\le v\le U\\ \sum _{i}({\hat{S}}_{i}^{T}{p}_{i}{y}_{i}+{R}_{i}^{T}{w}_{i})-\rho +\sigma =f\\ \rho ,\sigma \ge 0\\ {U}^{T}\sigma -{L}^{T}\rho -{f}^{T}v=\mathrm{0,}\end{array}\}$$

where *f* is the vector so that *f* ^*T*^ *v* = *v*_Growth_.

Suppose for each *I* that *S*′_*i*_ *v*′ = *α*_*i*_ *e* for some *α*_*I*_ ≠ 0. Then $\hat{v}$ satisfies *R*_*i*_ *v* = 0 for all *i*. Moreover, since $\hat{v}$ solves Eq. (13), the strong duality theorem of linear programming guarantees a solution to Eq. (19) with $v=\hat{v}$ and *w*_*i*_ = 0. Alternatively, if for some *I* we have *S*′_*i*_ *v*′ ≠ *α*_*i*_ *e* for all nonzero *α*_*I*_, then ${R}_{i}\hat{v}\ne 0$ and $\hat{v}$ is infeasible in Eq. (18). Hence, in this case ${\hat{S}}_{i}$ is not biologically possible for $\hat{v}$.□

### Computational stability of the RAMP framework

A straightforward implementation of RAMP (see Eq. (9)) as a computational model proved elusive. While we were able to determine optimal solutions in many situations for the tested genome-scale metabolic reconstructions, we were unable to find *consistent* achievement of optimality for the general RAMP problem with a wide variety of state-of-the-art algorithms to solve SOCPs. The computational challenge is likely caused by two characteristics specific to genome-scale metabolic reconstructed networks. First, a substantial number of variables are unsigned and, essentially, unbounded. Second, the FBA problems arising from genome-scale reconstructed metabolic networks are highly degenerate (a detailed discussion of this point is provided in ref. 47) and often have high-dimensional solution sets. These characteristics combine in a way that seems to hamper the underlying interior-point algorithms employed in native SOCP solvers. We also tried to use standard nonlinear reformulations that allowed us to experiment with nonlinear solvers, but again success was tepid at best.

Motivated by biological considerations, we therefore implemented a limited version of RAMP as a computational model. If the probabilistic variability of each constraint is restricted to a single coefficient, then the associated SOCP is linear. The linear constraint is constructed by, for example, assuming that the last coefficient of the *i*-th constraint is the sole random variable. Then for some *q*-vector *s* we have

$$\begin{array}{rcl}\Vert {R}_{i}v\Vert  & = & \Vert {\delta }_{1-\varepsilon }\sqrt{{P}_{i}}(I-e{p}_{i}^{T}){\hat{S}}_{i}v\Vert \\  & = & \Vert {\delta }_{1-\varepsilon }\sqrt{{P}_{i}}(I-e{p}_{i}^{T})[{S}_{i\mathrm{,1}}e,\ldots ,{S}_{i,(m-\mathrm{1)}}e,s]v\Vert \\  & = & \Vert {\delta }_{1-\varepsilon }\sqrt{{P}_{i}}[{S}_{i\mathrm{,1}}(e-e{p}_{i}^{T}e),\ldots ,{S}_{i,(m-\mathrm{1)}}(e-e{p}_{i}^{T}e),(I-e{p}_{i}^{T})s]v\Vert \\  & = & \Vert {\delta }_{1-\varepsilon }\sqrt{{P}_{i}}[\mathrm{0,}\ldots ,0,(I-e{p}_{i}^{T})s]v\Vert \\  & = & \Vert {\delta }_{1-\varepsilon }\sqrt{{P}_{i}}(I-e{p}_{i}^{T})s\Vert {v}_{n}.\end{array}$$

This calculation shows that if we restrict probabilistic variability to, for example, the coefficients of the growth reaction, then each of the SOCP constraints of the form

$$\Vert {R}_{i}v\Vert -{M}_{i}\le {p}_{i}^{T}{\hat{S}}_{i}v\le {M}_{i}-\Vert {R}_{i}v\Vert ,$$

can be re-written linearly as

20 $$\begin{array}{c}{p}_{i}^{T}{\hat{S}}_{i}v-\Vert {\delta }_{1-\varepsilon }\sqrt{{P}_{i}}(I-e{p}_{i}^{T}){\hat{S}}_{i,{\rm{Growth}}}\Vert {v}_{{\rm{Growth}}}\ge -{M}_{i}\\ {p}_{i}^{T}{\hat{S}}_{i}v+\Vert {\delta }_{1-\varepsilon }\sqrt{{P}_{i}}(I-e{p}_{i}^{T}){\hat{S}}_{i,{\rm{Growth}}}\Vert {v}_{{\rm{Growth}}}\le {M}_{i},\end{array}\}$$

where ${\hat{S}}_{i,{\rm{Growth}}}$ is the column of ${\hat{S}}_{i}$ containing the scenarios for the growth coefficient.

We choose to use the growth coefficients for two reasons. First, we have already noted that this is an empirically derived reaction that is based on the aggregate properties of a cell culture, and thus, the stoichiometric values are inherently associated with uncertainty. Furthermore, it is well known that biomass composition (and thus the values of the stoichiometric coefficients of the growth reaction) of a cellular culture is affected by nutrient conditions and the culture’s growth phase^48, 49^.

The linear re-formulation in Eq. (20) allows RAMP to be solved with standard linear simplex solvers, which proved to be computationally stable. All numerical work was conducted with the freeware GLPK or with the commercial solver Gurobi^©^, both of which worked well with the COBRA toolbox^37^.

### RAMP prediction of gene essentiality

We present two computational experiments to assist in assessing how stochastic variation affects RAMP’s ability to predict gene essentiality. The first experiment iteratively induces individual randomness in each of the growth coefficients, holding the others at their nominal value as stated in the model. The second experiment assumes simultaneous randomness in all growth coefficients. In all of the computational experiments, we used the *E*. *coli* genome-scale metabolic models iAF1260^24^ and iJO1366^25^ in a minimal nutrient environment consisting of (i) the models’ presets to emulate M9 medium, (ii) a maximal glucose uptake rate of 10 mmol/gDw/h, and (iii) unlimited oxygen uptake.

#### RAMP variation in a single growth coefficient

Here, we increase the level of uncertainty in a single growth coefficient in an iterative fashion, and we systematically go through all of the coefficients. The goal of this experiment is to gauge predictive ability against individual uncertainties in the growth equation, and we measure the predictive ability simply by comparing the number of essential genes with that predicted by FBA. If the predictive ability is stable for large percent variations of a single coefficient, then the cells of the culture could possibly be disparate in how they use the associated metabolite. If instead the predictive ability degrades with slight deviations, then the growth coefficient is more likely conserved across the culture.

The experiment uses *q* = 5 scenarios for each coefficient. The two most extreme scenarios multiply the coefficient by ±*σ* with probability *P*(3/2 ≤ *z*) = 0.0351, where *z* is standard normal variable. Two other scenarios multiply the coefficient by ±*σ*/2 with probability *P*(1/2 ≤ *z* ≤ 3/2) = 0.2389, and the other scenario leaves the coefficient unchanged with probability *P*(−1/2 ≤ *z* ≤ 1/2) = 0.4520. The value of *δ*_1−*ε*_ was 3, which meant that the probabilistic constraints were guaranteed with probability 0.9997.

The largest value of *σ* was calculated within an accuracy of 0.0001 and with a final value that allowed RAMP’s predictive accuracy to match that of FBA’s. Thus, starting from an initial value of *σ* = 0.0001, we (i) increased *σ* by a factor 2 until RAMP’s gene knockout predictions no longer matched that of FBA, and (ii) at this point, we initiated a binary search to identify the maximal value of the multiplier *σ*, which resulted in RAMP and FBA gene knockout predictions matching.

#### RAMP simultaneous variation in all growth coefficients

In this experiment we assumed simultaneous randomness in all growth coefficients. Four probabilistic models were used to assess RAMP’s overall sensitivity to stochastic variation, and each was compared against FBA’s gene knockout predictions as calculated by the COBRA toolbox (see Table 3 for results).

**Model 1**(**default**) Our default RAMP model assumes that the means of the growth coefficients are the values stated in the FBA model and that probabilistic variation is restricted to the first unspecified significant digit. Assuming ${10}^{-{d}_{i}}$ identifies this digit for the *i*-th growth coefficient, we further assume that each growth coefficient has the 5 scenarios in which it is perturbed by $\pm \eta \cdot {10}^{-{d}_{i}}$, where *η* is one of 0, 1, or 2. The probability of the scenario with no perturbation is *P*(−1/2 ≤ *z* ≤ 1/2) = 0.4520, with perturbation $\pm {10}^{-{d}_{i}}$ is *P*(1/2 ≤ *z* ≤ 3/2) = 0.2389, and with perturbation $\pm 2\cdot {10}^{-{d}_{i}}$ is *P*(3/2 ≤ *z*) = 0.0351, where *z* is a standard normal variable. Two examples of these scenarios are illustrated in Table 5. We choose to set *δ*_1−*ε*_ = 3, so that the probabilistic constraints have a 99.97% guarantee of satisfaction.

**Table 5:** The first column is the metabolite name from the iJO1366 model, and the second column contains the associated growth coefficient from the FBA model.

Metabolite	FBA Growth Coefficient	Scenario				
		1	2	3	4	5
2ohph[c]	−0.000223	−0.0002232	−0.0002231	−0.000223	−0.0002229	−0.0002228
adp[c]	53.95	53.948	53.949	53.95	53.951	53.952
probability		0.0351	0.2389	0.4520	0.2389	0.0351

The sign indicates whether the metabolite is an input (negative) or output of the growth reaction. The remaining columns are the scenarios for the default case along with their probabilities.

**Model 2** The second probabilistic model multiplies *η* in model 1 by the scalar *ρ* to assess how RAMP solutions adjust as the scenarios deviate from those of FBA. Eight tests with *ρ* = 2, 3,…, 9 were considered. Integers beyond 9 resulted in sign changes and were not considered. The probability scenarios from Model 1 were used, as well as *δ*_1−*ε*_ similarly being set to 3.

**Model 3** Since Model 2’s scaling by *ρ* disproportionately effects small growth coefficients, we compensate by replacing the scenarios of Model 1 with percentages of the growth coefficient itself. We choose different ranges for *σ*, using the scenarios $\mathrm{(1}\pm \,\sigma \cdot \eta )\cdot {\hat{S}}_{i,Growth}$, where *η* is one of 0, ±1/2, or ±1. The probabilities are unchanged from those in Model 1, and *δ*_1−*ε*_ is 3.

**Model 4** To assess how RAMP reacts to changes in the certainty of satisfying the probabilistic constraints, model 4 changes *δ*_1−*ε*_. All other model parameters are inherited from the default model.

In our testing of the predictive ability of these four computational RAMP models (for computational results, see Table 3), we used the short-hand notation given in Table 6 for our choice of combination of model and parameters.

**Table 6:** The choice of RAMP parameters for models 1–4 in our computational simulations.

Model 1	RAMP_1_	
Model 2	RAMP_2_	
Model 3	RAMP_3*a*_	0.001 ≤ *σ* ≤ 0.01
	RAMP_3*b*_	*σ* = 0.02
	RAMP_3*c*_	0.03 ≤ *σ* ≤ 0.3
Model 4	RAMP_4_	0.01 ≤ 2*ε* ≤ 0.9

#### Determination of bounds *M*_*i*_

The selection of *M*_*i*_ (see Eq. (9)) is an important parameter to decide. However, instead of imposing these bounds arbitrarily, these parameters are determined so that each RAMP model accurately returns the targeted, optimal growth rate. Let *γ* ^*^ be the optimal growth rate as calculated by the FBA model. The *M*_*i*_ values are calculated by solving the following optimization problem.

21 $$\begin{array}{c}{\rm{\min }}\,{\Vert M\Vert }_{1}\\ {\rm{subject}}\,{\rm{to}}\,\\ {v}_{Growth}\ge {\gamma }^{\ast }\\ \Vert {R}_{i}v\Vert -{M}_{i}\le {p}^{T}{\hat{S}}_{i}v\le {M}_{i}-\Vert {R}_{i}v\Vert ,\quad i=1,2,\ldots ,m\\ L\le v\le U\\ M\ge \mathrm{0,}\end{array}\}$$

where *M* is the vector whose *i*-th component is *M*_*i*_, and ${\Vert \cdot \Vert }_{1}$ is the *L*_1_ norm. Setting *M* to be the calculated optimal solution of Eq. (21) tightens the SOCP constraints while ensuring that the optimal growth rate is held at its desired value. We experimented with *L*_2_ and *L*_∞_ counterparts; however, the *L*_2_ norm suffered from inconsistent solves, and the *L*_∞_ norm overly relaxed constraints, which was not surprising.

### Comparison of RAMP and FBA with experimentally determined fluxes

We tested the ability of FBA and RAMP to agree with experimentally determined fluxes. For RAMP we solved,

22 $$\begin{array}{c}{\rm{\min }}\,{\Vert v-{v}_{EXP}\Vert }^{2}\\ {\rm{subject}}\,{\rm{to}}\\ {v}_{Growth}\ge \theta {\gamma }^{\ast }\\ \Vert {R}_{i}v\Vert -{M}_{i}\le {p}^{T}{\hat{S}}_{i}v\le {M}_{i}-\Vert {R}_{i}v\Vert ,\quad i=1,2,\ldots ,m\\ L\le v\le U.\end{array}\}$$

where *v*_*EXP*_ is the set of experimentally determined fluxes. The *M*_*i*_ values were calculated to achieve the FBA-optimal growth rate as noted in the previous section. The scenarios were those of Model 3 (RAMP_3_) with a 20% scaling of the growth equation, i.e. *σ* = 0.2. The (FBA) optimal growth rate *γ* ^*^ was scaled down by *θ* = 0.9 because the experimental fluxes were not guaranteed to coincide with optimized cultures. Note that Eq. (22) is an example of a non-linear (quadratic) objective within the RAMP formalism. Consequently, we determine a set of RAMP flux values that are as close to the experimental flux set as possible.

Likewise, the ability of FBA to realize the experimental fluxes was tested by solving the following adaptation of Eq. (2),

23 $$\begin{array}{c}{\rm{\min }}\,{\Vert v-{v}_{EXP}\Vert }^{2}\\ {\rm{subject}}\,{\rm{to}}\\ {v}_{Growth}\ge \theta {\gamma }^{\ast }\\ {S}^{\hat{c}}v=0\\ {L}^{\hat{c}}\le v\le {U}^{\hat{c}}.\end{array}\}$$

As with RAMP, *θ* was set to 0.9, and the solution was again as close as possible to *v*_*EXP*_.

## Electronic supplementary material

Supplementary information
