# Supplementary material for: Robust Analysis of Fluxes in Genome-Scale Metabolic Pathways
Source: Sci Rep. 2017 Mar 21;7:268. doi: 10.1038/s41598-017-00170-3 (PMC5427939; doi:10.1038/s41598-017-00170-3)
Supplement: Supplementary file 1 — Supplementary information [file 41598_2017_170_MOESM1_ESM.pdf]

Authors: MacGillivray, Ko, Gruber, Sawyer, Almaas & Holder
Title: Robust Analyis of Fluxes in Genome-Scale Metabolic Pathways

Reaction index	Experimental reaction
1	GLC + ATP -> G6P
2	G6P -> 6PG + NADPH
3	6PG -> P5P + CO2 + NADPH
4	G6P -> F6P
5	6PG -> T3P + PYR
6	F6P + ATP -> 2T3P
7	2P5P -> S7P + T3P
8	P5P + E4P -> F6P + T3P
9	S7P + T3P -> E4P + F6P
10	T3P -> PGA + ATP + NADH
11	PGA -> PEP
12	PEP -> PYR + ATP
13	PYR -> AcCoA + CO2 + NADH
14	OAA + AcCoA -> ICT
15	ICT -> OGA + CO2 + NADPH
16	OGA -> FUM + CO2 + 1.5ATP + 2NADH
17	FUM -> MAL
18	MAL -> OAA + NADH
19	MAL -> PYR + CO2 + NADH
20	OAA + ATP -> PEP + CO2
21	PEP + CO2 -> OAA
22	AcCoA -> AC + ATP
23	NADPH -> NADH
24	O2 + 2NADH -> 2P/OxATP
25	biomass
26	ICT + AcCoA -> MAL + FUM + NADH
27	DHAP -> PYR
28	ETH -> ETHxt
